# Tolerance thresholds underlie responses to DNA damage during germline development

**DOI:** 10.1101/gad.351701.124

**Published:** 2024-07-01

**Authors:** Gloria Jansen, Daniel Gebert, Tharini Ravindra Kumar, Emily Simmons, Sarah Murphy, Felipe Karam Teixeira

**Affiliations:** 1 Department of Genetics, University of Cambridge, Cambridge CB2 3EH, United Kingdom;; 2 Department of Physiology, Development, and Neuroscience, University of Cambridge, Cambridge CB2 3DY, United Kingdom

**Keywords:** CRISPR–Cas9, DNA damage, *Drosophila melanogaster*, *P*-element, double-strand break, genome integrity, germline development, hybrid dysgenesis, meiosis, transposable element

## Abstract

Selfish DNA modules like transposable elements (TEs) are particularly active in the germline, the lineage that passes genetic information across generations. New TE insertions can disrupt genes and impair the functionality and viability of germ cells. However, we found that in *P*–*M* hybrid dysgenesis in *Drosophila*, a sterility syndrome triggered by the *P*-element DNA transposon, germ cells harbor unexpectedly few new TE insertions despite accumulating DNA double-strand breaks (DSBs) and inducing cell cycle arrest. Using an engineered CRISPR–Cas9 system, we show that generating DSBs at silenced *P*-elements or other noncoding sequences is sufficient to induce germ cell loss independently of gene disruption. Indeed, we demonstrate that both developing and adult mitotic germ cells are sensitive to DSBs in a dosage-dependent manner. Following the mitotic-to-meiotic transition, however, germ cells become more tolerant to DSBs, completing oogenesis regardless of the accumulated genome damage. Our findings establish DNA damage tolerance thresholds as crucial safeguards of genome integrity during germline development.

## Abstract (precis)

In this study, Jansen et al. use a *Drosophila* dysgenesis model to demonstrate that tolerance thresholds to DNA damage constitute a crucial protective mechanism for maintaining germline genomic integrity and germ cell survival. They show that DNA double-strand breaks (DSBs) induced by *P*-element transposition cause mitotic germ cell loss through the activation of a CHK2 checkpoint and in a DSB dose-dependent manner, while postmitotic germ cells have higher DSB tolerance and progress further through oogenesis.

The germline is the cell lineage that is responsible for the inheritance of genetic information in multicellular eukaryotes. In most animal species, germ cells are set aside from somatic lineages during early embryonic development and follow a unique developmental program that culminates in the production of haploid gametes carrying the genetic material that is passed to the next generation (Dansereau and Lasko 2008). Due to its central role in genetic inheritance, the germline is known to be the battleground where genetic conflicts between selfish genetic elements, such as transposable elements (TEs), and the host genome take place (Doolittle and Sapienza 1980; Orgel and Crick 1980; Cosby et al. 2019). Excessive TE activity fulfils the selfish drive of TEs to increase in copy number per genome but can impair genome integrity and functioning, thereby threatening the faithful transmission of genetic information (Haig 2016; Bourque et al. 2018). Unsurprisingly, several host mechanisms exist to limit the activity of TEs in the germline, minimizing changes in the inherited genetic material (Malone et al. 2009; Molaro and Malik 2016; Ecco et al. 2017).

A textbook example of the detrimental effects of uncontrolled TE activity on the germline is provided by the *P*–*M* hybrid dysgenesis system in *Drosophila melanogaster.* In this system, excessive activity of the *P*-element transposon triggers chromosomal rearrangements and increased mutation rates, ultimately leading to sterility (Kidwell et al. 1977; Kidwell and Novy 1979; Bingham et al. 1982). This syndrome specifically affects the germline of the progeny arising from crosses between *P*-element-containing (*P*) and *P*-element-devoid (*M*) strains in a nonreciprocal manner (Kidwell et al. 1977; Bingham et al. 1982). In the progeny of females from *M* strains crossed to males from *P* strains, germ cells are lost during development, leading to fully sterile adults (known as dysgenic hybrids) (Kidwell and Novy 1979; Bingham et al. 1982; Teixeira et al. 2017). Progeny produced from the reciprocal cross between *P* strain females and *M* strain males (known as nondysgenic hybrids) are protected from *P*-element activity by a maternally inherited, small RNA-based TE silencing mechanism, and therefore are fully fertile (Aravin et al. 2007; Brennecke et al. 2008; Teixeira et al. 2017).

The *P*-element is a DNA transposon, a class of TEs that transpose by cut and paste mechanisms (Bingham et al. 1982; Engels et al. 1990; Kaufman and Rio 1992). Full-length *P*-elements (2.9 kb) encode a single polypeptide, *P*-transposase, which recognizes specific DNA sequence motifs just inside of the *P*-element 5′ and 3′ terminal inverted repeats (TIRs) (Kaufman et al. 1989). Upon binding, the *P*-transposase cleaves both DNA strands in a defined manner, excising the target DNA to form the strand transfer complex (Rio and Rubin 1988; Kaufman and Rio 1992; Beall and Rio 1997). This complex then mediates the integration of the excised DNA element at a different genomic locus, completing the transposition process (Beall and Rio 1998). Consequently, *P*-element transposition leads to sequence changes at both the excision and insertion loci (Bellen et al. 2004, 2011).

The model for how *P*-element activity leads to germ cell loss during dysgenesis postulates that mutagenesis and gene disruption, caused by new *P*-element insertions into coding regions, ultimately impair cell functioning and viability (Griffiths et al. 2000). However, given that the diploid *Drosophila* genome is largely haplosufficient (Lindsley et al. 1972), it has been challenging to reconcile how new *P*-element insertions, which affect only one of two alleles (Engels et al. 1990), could reproducibly affect germ cell function. Moreover, since *P*-elements are known to preferentially insert into noncoding regions, including promoters and regions overlapping origins of replication (Spradling et al. 2011), it remains unclear how random new insertions can lead to highly penetrant, lethal mutations. It has also been proposed that DNA damage caused by uncontrolled *P*-element transposition could lead to the mobilization of other TE families, creating cumulative threats to genome integrity (Khurana et al. 2011). However, the fully penetrant germ cell death phenotype imposes challenges to precisely determining the rates and the genomic sites of new *P*-element insertions in dysgenic germ cells. As such, this model remains mostly untested.

Despite considerable work dissecting the host silencing pathways controlling TE expression in the germline (Malone et al. 2009; Molaro and Malik 2016; Ecco et al. 2017), the impact that active TEs that have evaded silencing can have on germ cells remains poorly understood. Here, using *P*–*M* hybrid dysgenesis as a model, we show that excessive TE activity in embryonic germ cells leads to the accumulation of DSBs and persistent cell cycle arrest prior to the fully penetrant germ cell loss phenotype observed in early larval stages. Using FACS sorting coupled with single-cell, whole-genome DNA sequencing, we found that dysgenic embryonic germ cells acquire surprisingly few new *P*-element insertions and, in this aspect, are indistinguishable from nondysgenic PGCs. Given this, we tested whether inducing DNA damage at endogenously silenced *P*-elements, which mimics the excision step of *P*-element transposition, can by itself elicit germ cell loss. Using an engineered, Cas9-based transgenic system to inflict dosage- and sequence-specific DSBs at *P*-elements or at other noncoding sequences of the genome, we demonstrated that embryonic germ cells as well as mitotically dividing adult germ cells are sensitive to DSBs in a dosage-dependent manner. In contrast, once germ cells have completed mitotic cycles and acquired programmed DSBs during meiotic recombination, they become tolerant to DSBs, with oogenesis proceeding despite the accumulation of high levels of DNA damage. Taken together, our findings suggest that predefined DNA damage tolerance thresholds in the developing germline form a selective barrier that can shape transposon proliferation strategies.

## Results

### PGCs accumulate DSBs and fail to re-enter the cell cycle prior to cell death during *P*–*M* hybrid dysgenesis

**Figure 1.**
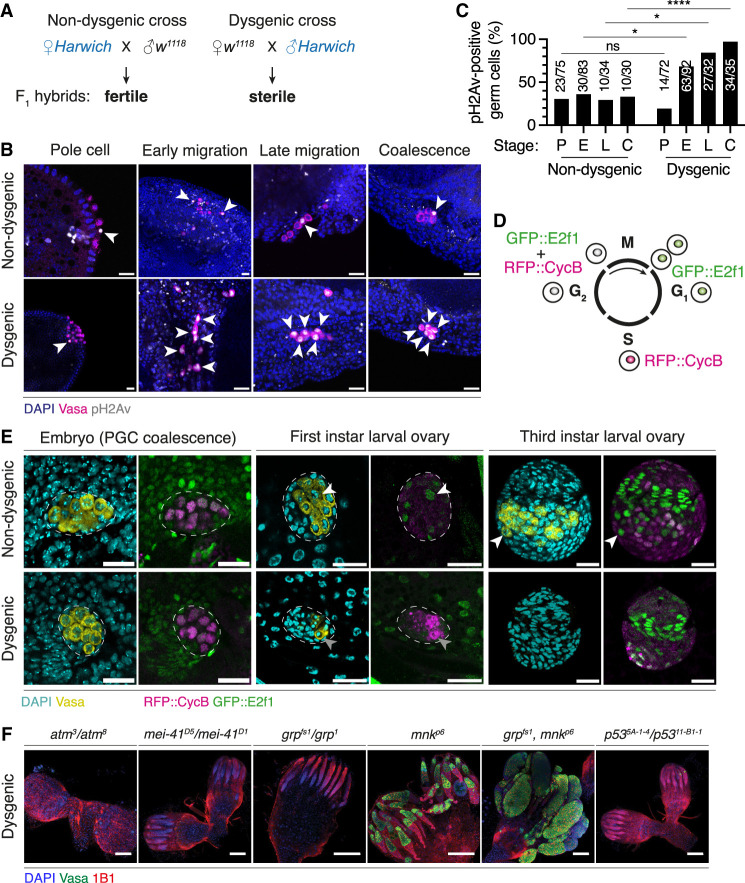
PGCs accumulate DSBs and undergo sustained cell cycle arrest during *P–M* hybrid dysgenesis. (*A*) *P*–*M* hybrid dysgenesis crossing scheme. (*B*) Dysgenic and nondysgenic hybrid progeny at four successive stages of embryonic germline development, labeled with DAPI (nuclei; blue), Vasa (germline; magenta), and pH2Av (DSBs; gray). White arrowheads indicate pH2Av-positive PGCs. (*C*) Proportion of pH2Av-positive PGCs in dysgenic and nondysgenic hybrid embryos at each developmental stage. (P) Pole cell, (E) early migration, (L) late migration, (C) coalescence. Absolute PGC numbers are shown *above* the bars. (ns) *P* > 0.05, (*) *P* ≤ 0.05, (****) *P* ≤ 0.0001; unpaired *t*-test. (*D*) Schematic showing Fly-FUCCI fluorescent readout at each cell cycle stage. (*E*) Dysgenic and nondysgenic hybrid progeny expressing Fly-FUCCI at three developmental stages (embryonic PGC coalescence and first and third instar larva), labeled with DAPI (cyan) and Vasa (yellow) in the *left* panels and with RFP (RFP::CycB; magenta) and GFP (GFP::E2f1; green) in the *right* panels. Dashed lines outline gonads within embryonic or larval tissue. PGCs that re-entered the cell cycle (white arrowheads) or remained arrested (gray arrowheads) are indicated. (*F*) Adult ovaries from dysgenic progeny carrying mutations in *atm*, *mei-41*, *grp*, *mnk*, *grp* and *mnk*, or *p53* (in a homozygous or transheterozygous state), labeled with DAPI (blue), Vasa (green), and 1B1 (somatic cells and spectrosomes; red). Scale bars, 100 μm.

In dysgenic hybrids, germline development proceeds normally during embryogenesis, but PGC numbers decrease from the first instar larval stage (Teixeira et al. 2017). To investigate the levels of DNA damage in dysgenic and nondysgenic PGCs prior to germ cell loss, we performed antibody staining against the phosphorylated histone H2A variant (pH2Av), a readout of DSBs (Madigan et al. 2002), at successive stages of embryonic development (Fig. 1A–C). At the pole cell stage, ∼20%–30% of both dysgenic and nondysgenic PGCs were positive for pH2Av. However, following germline zygotic genome activation (ZGA) at ∼4 h after egg laying (Van Doren et al. 1998), an increasing number of dysgenic PGCs accumulated strong pH2Av signal, with ∼70% of dysgenic PGCs positive for pH2Av at the early migration stage and >95% positive for pH2Av at gonadal coalescence. In contrast, the proportion of nondysgenic PGCs showing strong pH2Av signal stayed constant at ∼20%–30% during these stages.

Cell cycle arrest is a conserved response to genome damage (Xu et al. 2001; Melo and Toczyski 2002; Shim et al. 2014). To investigate cell cycle dynamics of PGCs during hybrid dysgenesis, we introduced the Fly-FUCCI cell cycle indicator system into the *P*-element-containing (*Harwich*) and *P*-element-devoid (*w*^*1118*^ or “*white*”) fly strains (Fig. 1D; Zielke et al. 2014). Fly-FUCCI relies on fluorescently tagged, ubiquitously expressed protein reporters whose activity provides a fluorescent readout of the distinct phases of the cell cycle in vivo. By crossing these strains reciprocally, we produced dysgenic and nondysgenic progeny and used confocal microscopy to determine the cell cycle phase of individual PGCs during embryonic and early larval development. In wild-type female flies, PGCs are known to exit the cell cycle upon their formation at the posterior pole of the embryo and remain stalled in G_2_ phase for a period of 16–18 h (Su et al. 1998). A few hours before larval hatching, PGCs progressively re-enter the cell cycle, with robust cycling activity only being observed during larval stages (Williamson and Lehmann 1996). Quantification of RFP (RFP::CycB; S/G_2_ phase) and GFP (GFP::E2f1; M/G_1_/G_2_ phase) signal in PGCs relative to somatic epidermal cells, which are known to be in G_1_ phase (Knoblich et al. 1994), indicated that both nondysgenic and dysgenic PGCs were arrested in G_2_ phase prior to embryonic gonadal coalescence (Fig. 1E; Supplemental Fig. S1A,B). However, while nondysgenic PGCs re-entered the cell cycle in early larval stages as shown by the loss of RFP::CycB signal, marking their asynchronous transition into M/G_1_ phase, all dysgenic PGCs remained arrested in G_2_ phase and failed to re-enter the cell cycle (Fig. 1E; Supplemental Fig. S1C,D). Together, these analyses indicate that DSBs induced following ZGA are associated with sustained cell cycle arrest and failure to re-enter the cell cycle and thus represent the earliest signatures of PGC loss during dysgenesis.

### Chk2, but not Chk1, triggers the germline checkpoint in a p53-independent manner

DNA damage responses are mediated by the kinases ataxia telangiectasia-mutated (ATM; encoded by *atm*/*tefu* in *Drosophila*) and ataxia telangiectasia and Rad3-related (ATR; *mei-41*) (Brodsky et al. 2004). To determine whether ATM or ATR is individually required for triggering the process leading to germ cell loss in dysgenesis, we established dysgenic and nondysgenic crosses between “*white*” and *Harwich* lines carrying mutations in *atm*/*tefu* or *mei-41* genes and tested whether these mutations can suppress the *P*-element-induced germline loss phenotype. In the absence of *P*-elements, transheterozygous or homozygous mutants for these genes are viable and show normal ovary morphology (Brodsky et al. 2004). Likewise, nondysgenic progeny mutant for *atm*/*tefu* and *mei-41* were viable, and ovary morphology was not affected. However, antibody staining and confocal analyses on adult ovaries of the dysgenic F_1_ progeny revealed that *atm/tefu* and *mei-41* mutants were devoid of germ cells (Fig. 1F; Supplemental Fig. S1E,F). These results suggest that neither ATM nor ATR is individually responsible for triggering the germ cell loss phenotype in dysgenic progeny. In agreement with this, ovaries of dysgenic progeny carrying mutations for the ATR-interacting partner ATRIP (*mus304*) were devoid of germ cells (Supplemental Fig. S1E,F).

Canonically, the ATR and ATM kinases are responsible for activating the checkpoint kinases Chk1 (*grp*) and Chk2 (*mnk*) (Brodsky et al. 2004). Given the cell cycle arrest phenotype of dysgenic PGCs, we wondered whether mutations in either *grp* or *mnk* could suppress the germ cell loss phenotype observed in dysgenic progeny. Strikingly, we consistently observed the suppression of germ cell death in dysgenic ovaries of flies that were mutant for *mnk* (Chk2) but not *grp* (Chk1) (Fig. 1F; Supplemental Fig. S1E,F). Adult dysgenic ovaries of *mnk* mutants frequently contained a small number of germ cells that did not progress normally through oogenesis. When stained with the 1B1 antibody, most of these cells showed either dot-like spectrosomes or branched fusomes (Supplemental Fig. S1E), which are markers of undifferentiated or partially differentiated germ cells, respectively (Kirilly and Xie 2007). On the other hand, adult dysgenic ovaries of *grp* mutants were mostly devoid of germ cells. This functional separation is reminiscent of the Chk2-dependent and Chk1-independent checkpoint that is activated in response to unrepaired meiotic DSBs during oogenesis (Abdu et al. 2002).

Importantly, the frequency of adult dysgenic ovaries containing germ cells was similar between the *mnk* single mutant and *grp, mnk* double mutant, indicating that Chk2 is mostly responsible for mediating the DNA damage checkpoint leading to germ cell death during dysgenesis, as previously suggested (Fig. 1F; Supplemental Fig. S1F; Moon et al. 2018; Rangan et al. 2011). However, the number of germ cells in dysgenic ovaries of *grp, mnk* double mutants was frequently larger than what was observed in the *mnk* single mutant, although most of these cells did not progress through oogenesis and frequently accumulated as partially differentiated cells, as revealed by 1B1 staining (Supplemental Fig. S1E). This suggests that while Chk1 does not mediate dysgenic germ cell death, it may have a role in slowing down the proliferation of dysgenic germ cells. On the other hand, it is well established that Chk2-mediated cell death normally transduces through the cell death effector p53 (Brodsky et al. 2004). Strikingly, mutations in *p53* did not suppress the dysgenic germ cell loss phenotype (Fig. 1F; Supplemental Fig. S1E,F). Altogether, these results reveal that Chk2, but not Chk1, is responsible for triggering the germ cell death checkpoint during dysgenesis and that Chk2 does not act via p53 to promote germ cell death.

During normal female germline development, DSBs are formed during meiotic recombination (Hughes et al. 2018). In *Drosophila*, meiotic DSB formation is mediated by *mei-W68* (the homolog of the yeast *Spo11*) and *mei-P22* (Mehrotra and McKim 2006). Given the strong DSB signal that we observed in dysgenic PGCs (Fig. 1B), we tested whether *mei-W68* or *mei-P22* plays a role in triggering the germline checkpoint in dysgenesis by assessing whether mutations in these genes can individually suppress the dysgenic germ cell loss phenotype. In the absence of *P*-elements and in nondysgenic progeny, *mei-W68* and *mei-P22* mutants are viable and show normal ovary morphology (Klattenhoff et al. 2007). In dysgenic progeny, ovaries mutant for *mei-W68* or *mei-P22* were mostly devoid of germ cells (Supplemental Fig. S1E,F). Together, these results indicate that neither *mei-W68* nor *mei-P22* is required to trigger the checkpoint in dysgenic germ cells.

### PGCs acquire few new *P*-element insertions during hybrid dysgenesis

**Figure 2.**
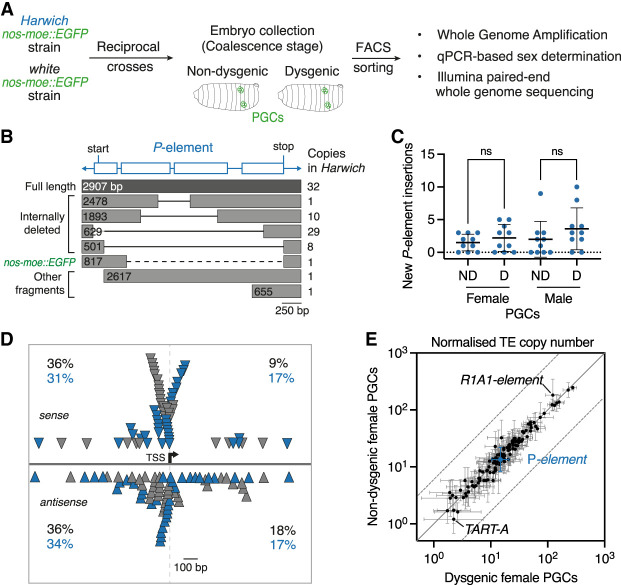
PGCs acquire few new *P*-element insertions during hybrid dysgenesis. (*A*) Experimental design for FACS sorting and whole-genome sequencing of single PGCs from dysgenic and nondysgenic hybrid embryos expressing the transgenic germline marker *nos-moe::EGFP*. (*B*) Genomic copy numbers and structures of *P*-elements present in the parental *Harwich* strain relative to the consensus sequence (blue; with positions of start and stop codons indicated). Black solid lines indicate internally deleted sequences. The *nos-moe::EGFP* transgene (dashed line) is flanked by *P*-element sequences. (*C*) Quantitation of new *P*-element insertions present in sorted PGCs but not present in *Harwich*. (ns) *P* > 0.05; unpaired *t*-test. (*D*) Map of *P*-element insertions existing in *Harwich* (gray triangles) and new insertions in PGCs (blue triangles) within 1 kb of the nearest host gene TSS. Proportion (percentage) of insertions in sense or antisense orientations (solid gray line) upstream or downstream (dashed gray line) relative to host genes are shown. (*E*) Genomic copy numbers of 126 *D. melanogaster* TE families in female dysgenic and nondysgenic PGCs (expressed in read base pairs per TE base pair divided by genomic coverage depth; log_10_). Error bars represent ±one standard deviation. The solid gray line represents perfect correlation, and dashed lines indicate fivefold change.

In dysgenic PGCs, activation of paternally inherited *P*-elements is hypothesized to induce high numbers of new *P*-element insertions (Griffiths et al. 2000; Khurana et al. 2011; Moon et al. 2018). To precisely determine the number and insertion sites of new *P*-element insertions in individual germ cells during embryogenesis, we isolated GFP-labeled PGCs from dysgenic and nondysgenic embryos by FACS and performed single-cell whole-genome DNA sequencing analysis (Fig. 2A). We isolated PGCs at late embryonic stages, which precede dysgenic germ cell death and during which >95% of dysgenic PGCs showed strong pH2Av signal and cell cycle arrest (Fig. 1B,C). Genomic DNA was extracted from individually sorted PGCs and amplified using the PCR-free, isothermal multiple displacement amplification technique (Dean et al. 2001). This procedure generated ∼5–23 μg of whole-genome amplified (WGA) DNA per sorted cell, with an average amplicon size of 10–18 kb (Supplemental Fig. S2A). Female and male sorted PGCs were distinguished using quantitative PCR to screen for the presence of the Y chromosome (Supplemental Fig. S2B). The WGA DNA of 40 individual PGCs was then used to generate DNA sequencing libraries (average genome coverage = 67×) (Supplemental Fig. S2C). These comprised 20 dysgenic and 20 nondysgenic PGCs, half of which were female and the other half of which were male.

To assess the impact of potential biases introduced by the whole-genome amplification procedure, we aligned reads to the reference genome and performed genome-wide coverage analysis over 10 kb windows. As expected, read coverage for single-cell samples presented more variability when compared with samples that were not subject to whole-genome amplification prior to library preparation (Supplemental Fig. S3). However, when taking the 40 individual cells into consideration, coverage variability was shown to be randomly distributed across the genome, as previously reported (Deleye et al. 2017). Overall, a high proportion (35%–95%) of the 10 kb windows (excluding chromosome 4 and the Y chromosome) displayed ≥10× read coverage in each analyzed cell, with 44%–97% of the genome displaying ≥5× read coverage.

To be able to discriminate new *P*-element insertions occurring in the genomes of isolated PGCs from those that were vertically transmitted, we characterized all insertions existing in the parental *Harwich* strain used to generate the sorted cells. To do so, we de novo assembled the genome of this strain using long read Nanopore sequencing (∼75× coverage) and short read Illumina sequencing (∼45× coverage) on bulk-extracted genomic DNA. Bioinformatic analysis revealed that the *Harwich* strain contained 32 full-length *P*-element insertions in addition to 48 insertions corresponding to nonautonomous, internally deleted elements (Fig. 2B; Supplemental Table S1; Karess and Rubin 1984; Laski et al. 1986; Rio and Rubin 1988; Teixeira et al. 2017). We also characterized *P*-element insertions in terms of their zygosity within the *Harwich* strain; i.e., whether they were present in one or both alleles at a given locus. To do so, we used the TE discovery tool TEMP on Illumina-generated reads (Zhuang et al. 2014). This tool detects new TE insertions through comparisons of genome sequencing data versus a reference genome. Since *P*-elements are absent from the reference *D. melanogaster* genome assembly (dm6), all insertions identified by TEMP represent *P*-element insertions present in the *Harwich* genome. Coverage frequencies calculated by TEMP, which served as a proxy for the zygosity of each insertion, revealed that 58% of insertions were homozygous or nearly homozygous (coverage frequency ≥0.7), while the remaining 42% of insertions were segregating at variable allele frequencies (average coverage frequency 0.3) (Supplemental Fig. S4A).

To determine the *P*-element discovery rate in the PGC genomes using TEMP, we focused on *P*-element insertions that are homozygous in the parental *Harwich* stock, as these insertions are expected to be present in every PGC. Taking genome coverage into account, homozygous insertions were detected at an average rate of 79% (Supplemental Fig. S4B). As expected, the detection rate was positively correlated with the frequency at which insertions were segregating in the *Harwich* parental strain (Supplemental Fig. S4B–D). Moreover, vertically transmitted insertions had coverage frequencies compatible with their expected heterozygote state in hybrid PGCs (Supplemental Fig. S4E). Overall, our analyses show that this approach is effective at detecting *P*-element insertions using DNA sequencing data obtained from single PGCs.

Using this approach, we then characterized new transposition events; i.e., *P*-element insertions found in individual PGCs that were not present in the parental *Harwich* genome. Focusing on female PGCs, which inherit one copy of each chromosome from the *Harwich* and “*white*” parental strains regardless of the direction of the cross, we found that dysgenic PGCs acquired between zero and five new *P*-element insertions per diploid genome (2.2 average), only 1.5-fold more new insertions than nondysgenic PGCs (1.5 average) (Fig. 2C; Supplemental Table S2). Male dysgenic and nondysgenic PGCs are not genetically identical due to the parent-of-origin inheritance of X and Y chromosomes. Despite this, and as observed for female PGCs, both dysgenic and nondysgenic male PGCs acquired few new insertions per cell (3.6 and 2.0 average, respectively). The read coverage of new *P*-element insertions was consistent with a heterozygous state, suggesting that these insertions are present in one of the two alleles (Supplemental Fig. S5A).

Next, we examined the genome annotation associated with the new *P*-element insertions in the PGC genomes. Similar to what was observed for *P*-element insertions present in the *Harwich* background, new *P*-element insertions were dispersed along the autosomes and X chromosome (Supplemental Fig. S5B). Based on chromatin state annotations of the reference genome, 60% of the new insertions were located in transcriptionally active euchromatic regions, while the remaining 40% were located in heterochromatic or transcriptionally repressed regions (Supplemental Table S2; Filion et al. 2010). New insertions were mostly located within promoters, 5′ untranslated regions (UTRs), and first introns of genes, confirming previous findings (Supplemental Table S2; Spradling et al. 2011). By mapping their position relative to the transcription start sites (TSSs) of host genes, we found that 75% of new insertions were within 1 kb of the closest TSS, mirroring what was found for insertions existing in the *Harwich* strain (Fig. 2D). As was previously observed for transgenic *P*-element insertions, both parental and new *P*-element insertions were also enriched in regions overlapping origins of replication (Supplemental Tables S1, S2; Spradling et al. 2011).

Aside from *P*-elements, we sought to determine whether other transposon families become activated in PGCs during *P*-element hybrid dysgenesis, as previously proposed (Khurana et al. 2011). Copy numbers of 126 TE families present in the *D. melanogaster* genome were highly similar between dysgenic and nondysgenic PGCs in both females and males (Fig. 2E; Supplemental Fig. S5C). This suggests that TE families other than the *P*-element are unlikely to increase in copy number in dysgenic PGCs or contribute to the germ cell loss phenotype, as previously suggested (Eggleston et al. 1988; Moon et al. 2018).

Overall and contrary to predictions based on the insertional mutagenesis model of hybrid dysgenesis (Griffiths et al. 2000; Khurana et al. 2011), our analysis shows that dysgenic and nondysgenic PGCs acquire similar, low numbers of new heterozygous *P*-element insertions mostly located within the promoters and introns of genes.

### PGCs are sensitive to DSBs at *P*-elements in a dosage-dependent manner

Our single-cell analysis revealed that dysgenic PGCs acquire few, scattered new *P*-element insertions, although, at the same developmental stage, virtually all dysgenic PGCs showed a strong accumulation of DSBs and cell cycle arrest (Fig. 1A–E). We hypothesized that this discrepancy could relate to the transposition mechanism used by *P*-elements, which induces DSBs at both the excision and insertion steps. In this context, excision of multiple *P*-element copies, such as those that are typically present in *P* strains, has the potential to induce high levels of DSBs (at least one per *P*-element copy) (Bingham et al. 1982). Notably, all full-length and internally deleted *P*-element insertions in the *Harwich* background are flanked by intact TIRs, which can be recognized by the *P*-transposase and cleaved during the excision step (Fig. 2B).

**Figure 3.**
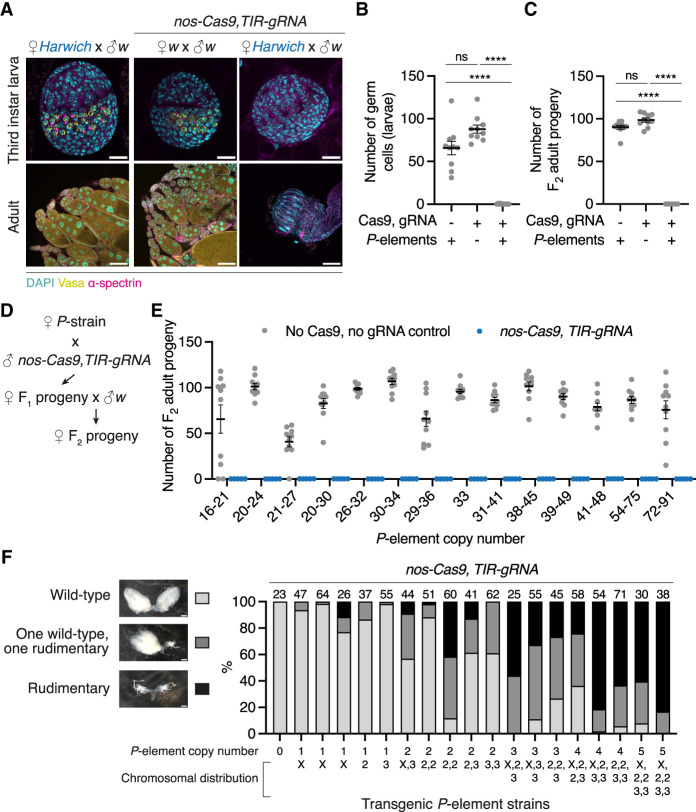
Inducing DSBs at silenced *P*-elements is sufficient to induce germ cell loss. (*A*) Third instar larval ovaries (*top* panel) and adult ovaries (*bottom* panel) from nondysgenic progeny lacking *nos-Cas9, TIR-gRNA* (*left* panels), progeny without *P*-elements and expressing *nos-Cas9, TIR-gRNA* (*middle* panels), and progeny with *P*-elements and expressing *nos-Cas9, TIR-gRNA* (*right* panels), labeled with DAPI (cyan), Vasa (yellow), and α-spectrin (cell borders; magenta). (*B*) The number of germ cells present in larval ovaries (gray data points; *n* = 10) from the F_1_ progeny shown in *A*. The black line indicates mean, and error bars indicate ±SEM. (*C*) Fertility of adult female progeny shown in *A* as determined by the number of F_2_ progeny originating from single F_1_ female crosses (*n* = 10). (****) *P* < 0.0001, (ns) *P* > 0.05; one-way ANOVA and Tukey's multiple comparisons test. (*D*) Crossing scheme used to test whether targeting Cas9 to *P*-elements in wild-derived isogenic *P*-strains, which have variable *P*-element content, modulates the penetrance of the germ cell loss phenotype observed in the *Harwich* background (shown in *A*). (*E*) Fertility of adult female progeny of the crosses in *D* in the presence (blue data points) or absence (gray data points) of *nos-Cas9, TIR-gRNA*, as measured by the number of F_2_ progeny originating from single F_1_ female crosses (*n* = 10). *P*-element copy number ranges represent estimates determined by DNA qPCR of 5′ and 3′ regions of the *P*-element. *P* < 0.01 for all pairwise comparisons; unpaired *t*-test. (*F*) Females from laboratory strains carrying between one and five transgenic *P*-element copies with varying chromosomal locations were crossed to *nos-Cas9, TIR-gRNA* males. Ovary morphology of the progeny was categorized as wild type (light gray), one wild-type and one rudimentary ovary (dark gray), or two rudimentary ovaries (black), as shown in representative bright-field images. The number of ovary pairs analyzed for each genotype is indicated *above* each bar. Scale bars: *A*, larva, 20 μm; *A*, adult, 100 μm; *F*, 100 μm.

To test whether DSBs at *P*-elements present in the genome of the *Harwich* strain are sufficient to elicit PGC loss, we generated a transgenic line expressing the CRISPR-associated protein 9 (Cas9) specifically in the germline in combination with a cassette expressing a *P*-element TIR-specific guide RNA (gRNA) (Supplemental Fig. S6). The Cas9–TIR gRNA transgene was inserted into the “*white*” genetic background, which is devoid of *P*-elements. As expected by the absence of targets for the Cas9–TIR gRNA effector in this background, transgenic strains were homozygous-viable, had normally developed ovaries, and were fully fertile (Fig. 3A,C). Targeting Cas9 to the *P*-element TIRs in nondysgenic progeny, in which *P*-elements are present but not active, enabled us to induce DSBs in a sequence-specific manner and thereby mimic the damage caused by *P*-element excision. In contrast to what was observed in the “*white*” background (or in nondysgenic control progeny lacking the Cas9–TIR gRNA transgene), when the Cas9–TIR gRNA transgene was introduced into nondysgenic progeny, flies were viable but completely lacked germ cells and were fully sterile (Fig. 3A,C). As we observed in hybrid dysgenesis, the germ cell loss phenotype induced by Cas9–TIR gRNA emerged during fly development, and most PGCs were lost by the third instar larval stage (Fig. 3A,B). These results suggest that inducing DSBs at resident *P*-elements, which are present in a heterozygous state, is sufficient to elicit germ cell loss at the same full penetrance observed in hybrid dysgenesis.

Given that the *Harwich* strain contains >80 *P*-elements (Supplemental Table S3), we asked whether a lower *P*-element copy number, and consequently a lower number of potential DSB sites, would impact the penetrance of the germline loss phenotype. Taking advantage of the *Drosophila* Genetic Reference Panel (DGRP) collection (Mackay et al. 2012), we selected a set of 14 wild-derived isogenic strains containing variable numbers of *P*-elements. First, we used publicly available estimates of *P*-element copy numbers that were based on analyses of sequencing data from strains in the DGRP collection (Rahman et al. 2015). We then confirmed *P*-element copy number in the subset of 14 selected DGRP stocks by quantitative PCR using primers corresponding to the 5′ and 3′ ends as well as an internal sequence only present in full-length copies (Supplemental Table S3). This analysis showed that the strains contained between ∼16 and ∼91 *P*-element copies. Females from each DGRP strain were individually crossed to males carrying Cas9–TIR gRNA in the *P*-element-devoid “*white*” background to generate nondysgenic progeny containing both *P*-elements and Cas9–TIR gRNA (Fig. 3D). Surprisingly, nondysgenic progeny for all these crosses were fully sterile (Fig. 3E), indicating that tolerance for DSBs at heterozygous *P*-element insertions in the germline may be lower than the copy number range naturally occurring in wild strains.

Outside of their occurrence in natural strains, *P*-elements have been successfully used in the last 40 years to generate transgenic *Drosophila* laboratory strains (otherwise devoid of *P*-elements) (Rubin and Spradling 1982; Spradling and Rubin 1982). For transgenesis, exogenously provided *P*-transposase was coinjected into laboratory strain embryos with donor vectors containing the transgenic sequences of interest, which are flanked by *P*-element TIRs (Rubin and Spradling 1983). This process resulted in the random integration of TIR-flanked constructs into the host genome. Taking advantage of the extensive collections of publicly available and previously characterized transgenic strains, we used genetic crosses to combine *P*-element-derived transgenes on different chromosomes, resulting in strains carrying between one and five transgene copies and therefore much lower numbers of Cas9–TIR gRNA targets compared with wild strains (Supplemental Table S3). We individually crossed females from the transgenic laboratory strains to “*white*” males carrying Cas9–TIR gRNA and scored ovary morphology in the F_1_ progeny as a proxy for germline loss. Our analysis showed that the number of rudimentary ovaries, which lacked germ cells, was positively correlated with the number of transgenic *P*-elements present in the parental genome (Fig. 3F). Flies carrying one to two transgene copies were found to be mostly fertile, while rudimentary ovaries were observed in flies carrying more than two transgene copies. In the presence of five *P*-element-derived transgenes, ovaries were mostly rudimentary, indicating that DSBs at the TIRs of as few as five heterozygous *P*-elements were sufficient to induce severe germ cell loss. Taken together, our results indicate that mimicking the DNA damage formed during *P*-element excision is sufficient to elicit germ cell loss at the same full penetrance observed in dysgenesis and that the number of DSBs that PGCs tolerate is low.

### PGCs are sensitive to DSB dosage independent of *P*-elements

**Figure 4.**
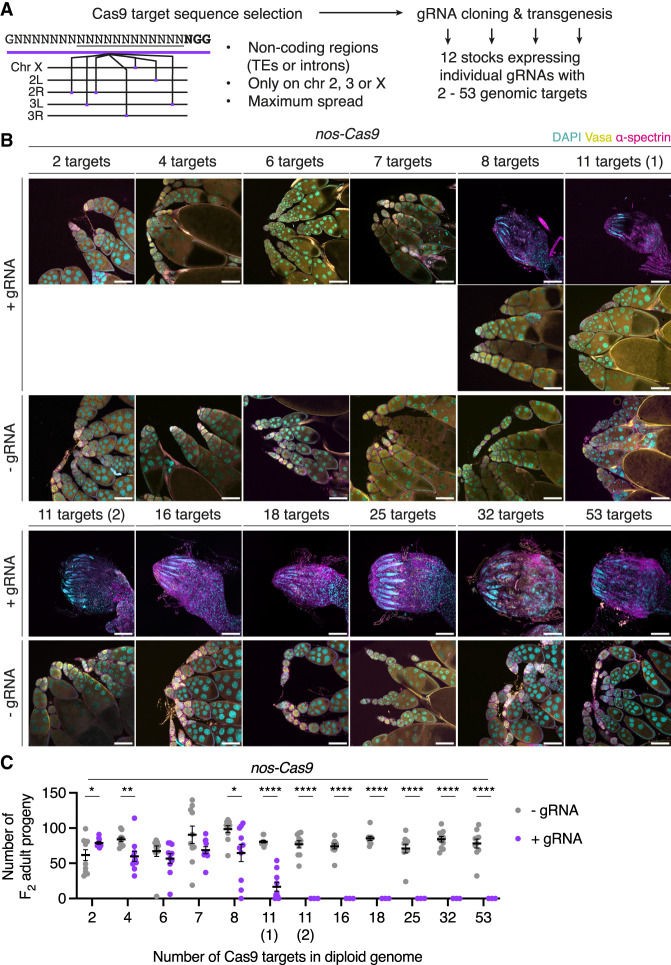
PGCs are sensitive to DSBs in a dosage-dependent manner. (*A*) Cas9-based approach to systematically test DNA damage tolerance in the germline. gRNA sequences with multiple genomic copy numbers within TEs or other noncoding regions on the second, third, or X chromosome were identified in the genomes of strains expressing Cas9 and gRNA transgenes. Individual gRNAs were separately cloned into an *att*B-containing vector for φC31-mediated transgenesis, generating 12 lines expressing gRNAs with specific numbers of genomic targets in the diploid genome. (*B*) Ovaries of adult F_1_ progeny from individual crosses of gRNA-expressing females and *nos-Cas9* males, labeled with DAPI (cyan), Vasa (yellow), and α-spectrin (magenta). Sibling progeny lacking the gRNA transgene were used as controls (−gRNA). Variable ovary morphology (wild type and rudimentary) was observed in the presence of eight-target gRNA and one of two gRNAs with 11 targets [11 targets (1)]. (*C*) Fertility of the F_1_ progeny in *B* in the presence (purple data points) or absence (gray data points) of gRNA, as measured by the number of F_2_ progeny originating from single F_1_ female crosses (*n* = 10). Pearson coefficient (+gRNA), *r* = –0.68. (*) *P* ≤ 0.05, (**) *P* ≤ 0.01, (****) *P* ≤ 0.0001; unpaired *t*-test. The black line indicates mean, and error bars indicate ±SEM. Scale bars, 100 μm.

Given our results suggesting that PGCs tolerate few DSBs at *P*-elements, we sought to test whether this effect is a more general feature of germ cells independent of specific target sequences (*P*-element or non-*P*-element). For this purpose, we generated whole-genome assemblies using long read Nanopore (∼70× coverage) and short read Illumina (∼50× coverage) sequencing data from a strain expressing Cas9 in the germline (*nos-Cas9*), a strain containing a docking site for gRNA-expressing transgenes (*nos-int;attP2*), and a strain used to generate balanced transgenic lines (*w;TM2/TM6*). In these assemblies, we computationally searched for gRNA target sequences present in specific genomic copy numbers (Fig. 4A). To avoid potential confounding effects that could arise by targeting Cas9 to coding sequences, promoters, and UTRs, which may affect cell viability independently of the DSB dosage effect, we devised a tool to identify target sequences only within transposons or other noncoding sequences. We further excluded sequences present in multiple copies in close proximity to avoid inducing clustered DSBs, which could lead to rearrangements or large indels. We then performed transgenesis of these gRNA sequences (expressed ubiquitously under the control of the U6 promoter) into the strain containing a transgene docking site (*nos-int;attP2*). In total, we established 12 transgenic lines expressing individual gRNAs targeting between two and 53 sites in the diploid genome (Supplemental Table S4).

To induce DSBs at these sites in PGCs, we individually crossed females from the gRNA-expressing lines to males from the strain expressing Cas9 in the germline (*nos-Cas9*) and assessed ovary morphology of the female progeny. While ovary morphology resembled the wild type in the presence of up to seven Cas9 target sites in the diploid genome, increasing numbers of rudimentary ovaries were observed in females carrying gRNAs targeting eight and 11 sites (Fig. 4B). All ovaries from females carrying gRNAs targeting >11 sites were found to be rudimentary and completely lacked germ cells, as shown by the absence of the germline marker Vasa. We assessed the effect of the germline loss on fertility and found that it was negatively correlated with the number of Cas9 target sites (Fig. 4C). Targeting catalytically inactive dead Cas9 to the same sites in the germline did not affect ovary morphology, showing that Cas9-mediated DSBs, rather than Cas9's presence or binding, led to germline loss (Supplemental Fig. S7A). Together, these results indicate that germ cells are sensitive to DNA damage levels.

We sought to validate how efficiently the Cas9–gRNA system induced DSBs. If the system is efficient, we reasoned that targeting Cas9 to a single site in the coding sequence (CDS) of an essential gene would suffice to induce complete germ cell loss. To test this, we designed individual gRNAs targeting the CDSs of three ribosomal protein (RP) genes and established transgenic stocks. Crosses between RP-gRNA and *nos-Cas9* strains produced viable female progeny with rudimentary ovaries that lacked germ cells (Supplemental Fig. S7B). In a small proportion of ovaries, germaria and early egg chambers were observed, but egg chambers degenerated at mid-oogenesis stages, coinciding with the nutritional checkpoint that precedes vitellogenesis (Drummond-Barbosa and Spradling 2001). Accordingly, these flies were mostly sterile (Supplemental Fig. S7C). Together, our analysis shows that the Cas9 system can reproducibly induce DSBs at target sites.

To further assess the efficiency of Cas9-mediated DSB formation at the molecular level, we crossed gRNA strains with target numbers below the germ cell loss-inducing threshold (<11) to *nos-Cas9* males to obtain progeny with Cas9-edited germ cells. By crossing this F_1_ progeny to wild-type “*white*” males, we obtained F_2_ flies that we expected to harbor DSB repair products at target sites. We extracted genomic DNA from 10 individual F_2_ progeny for each tested gRNA and amplified individual target sites and flanking regions by PCR. Sequencing analysis revealed that, on average, 92% of target sites were edited in the F_2_ progeny (Supplemental Fig. S7D). Interestingly, in most cases, both alleles contained small indels around the target site (Supplemental Fig. S7E), suggesting that maternally deposited Cas9–gRNA complexes continued to target paternally inherited “*white*” chromosomes in the F_2_ progeny and that repair most likely occurred by nonhomologous end joining (Lieber 2010). Taken together, our molecular analysis confirms that the Cas9–gRNA system is highly efficient at inducing DSBs at its target sites.

### Mitotically dividing adult germ cells are sensitive to DSB levels

During larval development, PGCs mature into germline stem cells (GSCs) (Dansereau and Lasko 2008). In the adult ovary, GSCs continuously divide to support the production of eggs. Asymmetric GSC divisions generate differentiating daughters, which are excluded from the stem niche and thereafter undergo four mitotic cell divisions with incomplete cytokinesis to form 16 cell germline cysts (Kirilly and Xie 2007). Meiosis begins once mitotic divisions are completed, and meiotic recombination, which involves the programmed formation and repair of DSBs, occurs at the 16 cell cyst stage while cysts are progressing through the germarium (Hughes et al. 2018). Meiotic DSBs are repaired before the 16 cell cyst exits the germarium and develops into an egg chamber containing one oocyte and 15 nurse cells, which will then further develop into a mature egg (Mehrotra and McKim 2006). During the meiotic DSB repair cycle, each germ cell acquires 20–24 DSBs (Mehrotra and McKim 2006). As such, the number of meiotic DSBs acquired by adult germ cells during meiotic recombination is around double the number of Cas9 targets that we found was sufficient to result in complete germ cell loss during development (Fig. 4B).

**Figure 5.**
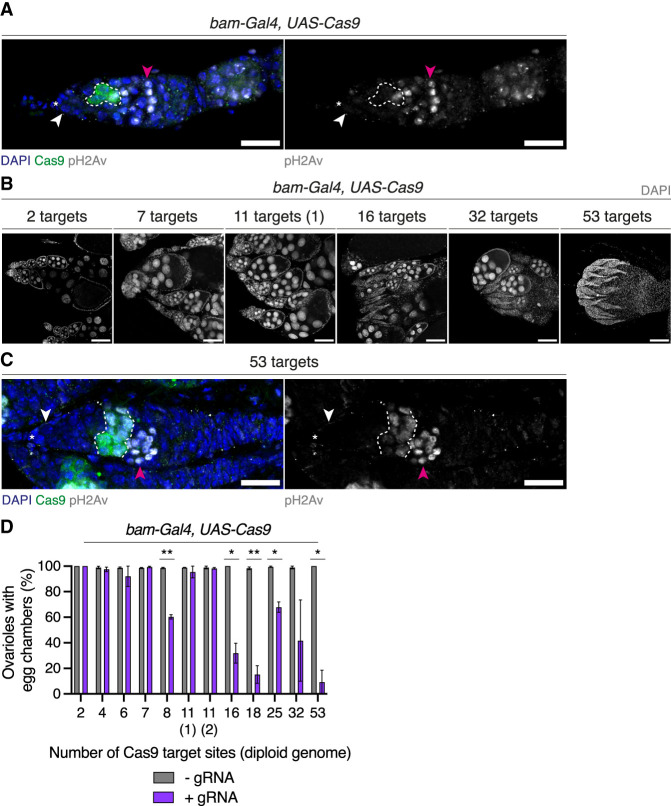
Dosage-dependent sensitivity to DSBs in premeiotic adult germ cells. (*A*) Germarium from a strain expressing Cas9 in the *bam* domain, labeled with DAPI (blue) and Cas9 (green) in the *left* panel and with pH2Av (gray) in the *right* panel. Asterisks indicate the GSC niche, white arrowheads indicate GSCs, and magenta arrowheads indicate meiotic germ cells. The dashed line demarcates the Cas9 expression domain within 2 to 8 cell cysts. (*B*) Adult ovaries of F_1_ progeny from individual crosses between gRNA-expressing females and *bam-Gal4, UAS-Cas9* males, labeled with DAPI (gray). (*C*). Germarium of F_1_ female in which Cas9 was targeted to 53 target sites in the *bam* domain, labeled as in *A*. Asterisks, arrowheads, and dashed lines are as in *A*. (*D*) Proportion of ovarioles containing egg chambers in *B* in the presence (purple bars) or absence (gray bars) of gRNA. Pearson coefficient (+gRNA), *r* = –0.76. Error bars indicate ±SEM. *n* > 150 ovarioles per genotype from two independent replicates. (*) *P* ≤ 0.05, (**) *P* ≤ 0.01; unpaired *t*-test. Scale bars: *A*,*C*, 20 μm; *B*, 100 μm.

To investigate tolerance to DSBs in the adult germline prior to meiotic DSB formation, we expressed Cas9 in the *bam* domain (Fig. 5A). The differentiation factor *bam* is expressed in a narrow developmental window during the cystoblast to 8 cell cyst stages in the germarium, when cells are still dividing mitotically and are yet to enter meiosis (McKearin and Spradling 1990; Mehrotra and McKim 2006). We used our previously characterized panel of transgenic gRNA lines targeting different numbers of genomic sites in combination with *bam-Gal4*-driven expression of *UAS-Cas9* to induce different numbers of DSBs in mitotically dividing germ cells in the adult ovary. Immunofluorescence microscopy analysis revealed that targeting up to 11 sites resulted in ovaries with wild-type morphology. However, when we targeted Cas9 to >11 sites, ovarioles contained germaria but had aberrant egg chamber morphology (Fig. 5B). Staining for DSBs with the pH2Av antibody showed that strong DSB signal was present in cysts within the *bam* expression domain and in more posteriorly localized, further developed cysts, with the signal in these cysts likely representing meiotic DSBs (Fig. 5C). We quantified the number of ovarioles containing egg chambers and found that this was negatively correlated with the number of Cas9 target sites (Fig. 5D). This suggests that, like PGCs, mitotically dividing adult germ cells are sensitive to DSBs in a dosage-dependent manner and that their tolerance threshold lies below the number of DSBs induced during meiotic recombination.

### Postmitotic germ cells in the adult ovary are resilient to DNA damage

To determine whether developing germ cells are equally sensitive to DSB levels after the meiotic DSB-repair cycle, we expressed Cas9 in the *TOsk* domain (ElMaghraby et al. 2022). The *TOsk-Gal4* driver is first expressed in postmitotic germ cells (16 cell cysts) shortly after meiotic DSBs are first formed, and its expression persists throughout the remainder of oogenesis until mature eggs are formed. At the 16 cell cyst stage, the oocyte enters meiotic prophase I, where it remains until oogenesis is completed, while the nurse cells enter a specialized program of continuous, rapid cell cycles between S and G_2_ phase without cell divisions (known as endocycles), becoming polyploid to synthesize the materials required for oocyte growth (Hughes et al. 2018; Hinnant et al. 2020).

**Figure 6.**
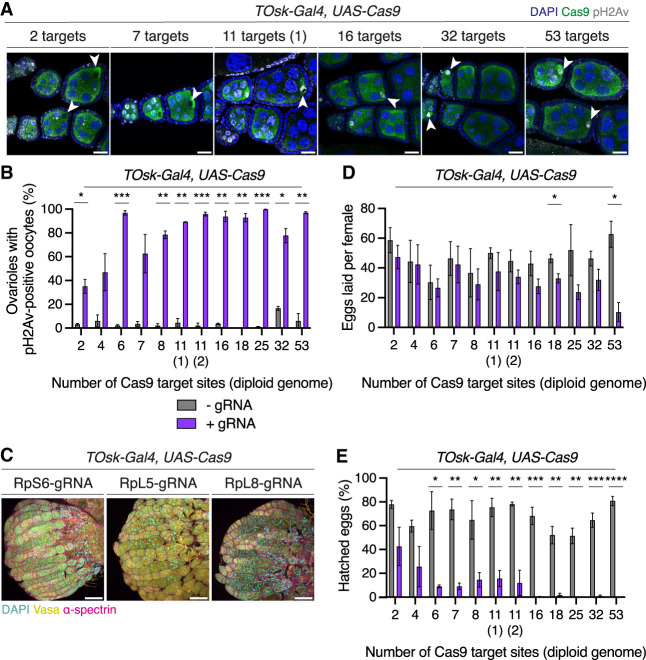
High DSB levels are tolerated following the mitotic-to-meiotic transition. (*A*) Mid-stage egg chambers of F_1_ progeny from individual crosses between gRNA-expressing females and *TOsk-Gal4, UAS-Cas9* males, labeled with DAPI (blue), Cas9 (green), and pH2Av (gray). White arrowheads indicate oocytes. (*B*) Proportion of ovarioles with pH2Av-positive oocytes in mid-stage egg chambers shown in *A* in the presence (purple bars) or absence (gray bars) of gRNA. *n* ≥ 90 ovarioles per genotype from two independent replicates. Pearson coefficient (+gRNA), *r* = 0.49. (*C*) Adult ovaries of F_1_ progeny from individual crosses between RP-gRNA-expressing females and males expressing Cas9 in the *TOsk* domain, labeled with DAPI (cyan), Vasa (yellow), and α-spectrin (magenta). (*D*) Average number of eggs laid per F_1_ female (*n* = 10, in three independent replicates) from the crosses in *A*. Pearson coefficient (+gRNA), *r* = –0.76. (*E*) Proportion of eggs laid by F_1_ females in *C* that hatched. Pearson coefficient (+gRNA), *r* = –0.64. Error bars indicate ±SEM. (*) *P* ≤ 0.05, (**) *P* ≤ 0.01, (***) *P* ≤ 0.001, (****) *P* < 0.0001; unpaired *t*-test. Scale bars, 100 μm.

To induce different amounts of DSBs in postmitotic germ cells, we used the panel of transgenic gRNA lines targeting different numbers of genomic sites in combination with a line expressing Cas9 in the *TOsk* domain (Supplemental Fig. S8A). In contrast to PGCs and mitotically dividing adult germ cells and regardless of the number of Cas9 targets (up to as many as 53), all adult female flies had morphologically wild-type ovaries that contained mature eggs (Supplemental Fig. S8B). We performed antibody staining against pH2Av to determine whether DSB signal was present in these ovaries. As in wild-type ovaries, in both the absence and presence of gRNAs, nurse cell nuclei of early egg chambers contained pH2Av signal, which mostly disappeared from egg chambers stage 4 onward (Fig. 6A; Supplemental Fig. S8A). On the other hand, and only in the presence of Cas9 and gRNAs, strong pH2Av signal was observed in oocyte nuclei of mid-stage egg chambers. Most ovarioles (62%–99%) contained pH2Av-positive oocytes in the presence of gRNA targeting six to 53 sites (Fig. 6B). These results suggest that, in contrast to PGCs and mitotically dividing adult germ cells, germline development can proceed despite persistent DSBs in oocyte nuclei.

To confirm that the wild-type ovary morphology that we observed was not due to the strength of expression of the *TOsk-Gal4* driver, we used our previously established RP-gRNA lines to target Cas9 to a single site within the CDSs of essential genes and assessed whether this induces germ cell loss in the *TOsk-Gal4* domain. Microscopy analyses revealed that while ovaries contained wild-type germaria and early egg chambers, mid-oogenesis egg chambers had an aberrant, degenerated morphology, and ovaries did not contain any mature eggs (Fig. 6C). The high penetrance of these morphological aberrations indicates that the *TOsk-Gal4*-driven expression of Cas9 was sufficient to induce efficient targeting in developing egg chambers, including the polyploid nurse cell nuclei.

Given the strong pH2Av signal present in flies carrying gRNA and expressing Cas9 postmitotically, we examined the morphology of the karyosome, a structure formed by the oocyte chromosomes following meiotic recombination. Wild-type karyosome morphology is known to change as a consequence of unrepaired meiotic DSBs and irradiation-induced DNA damage (Ghabrial et al. 1998; Abdu et al. 2002; Shim et al. 2014). In the presence of gRNAs targeting seven or fewer genomic sites, karyosomes consistently presented spherical morphology resembling that observed in control flies lacking gRNA (Supplemental Fig. S8C). In contrast, in the presence of gRNAs targeting more than seven genomic sites, karyosomes showed several aberrant morphologies, with most appearing oval and others appearing fragmented. These results indicate that inducing high levels of DSBs in postmitotic germ cells results in aberrant oocyte chromosome clustering.

Regardless of the number of noncoding sites targeted by Cas9 in postmitotic germ cells, the resulting ovaries contained eggs. To determine egg laying and hatching rates, we crossed the F_1_ progeny expressing *TOsk-Gal4* and *UAS-Cas9* (with or without gRNAs) to wild-type “*white*” males. While the number of eggs laid in the presence or absence of gRNAs was comparable, hatching rates were reduced by 34%–80% in the presence of gRNAs relative to controls (Fig. 6D,E). We allowed eggs to develop for 16 h, at which point PGCs have normally coalesced into embryonic gonads, and performed microscopy analysis on aged embryos. In the presence of gRNAs targeting <11 sites, embryos reached PGC coalescence stages and had wild-type morphology (Supplemental Fig. S8D). However, in the presence of gRNAs targeting >11 sites, embryos showed abnormal morphology, and PGCs were mislocalized and did not coalesce to form gonads. While it is difficult to untangle whether the impact on the development of the subsequent generation is due to the damage caused during oogenesis or the likely persistence of Cas9 activity due to maternal deposition, these results indicate that postmitotic germ cells can tolerate high levels of DSBs and yet produce viable progeny.

### DNA damage tolerance varies between mitotic and postmitotic somatic domains

**Figure 7.**
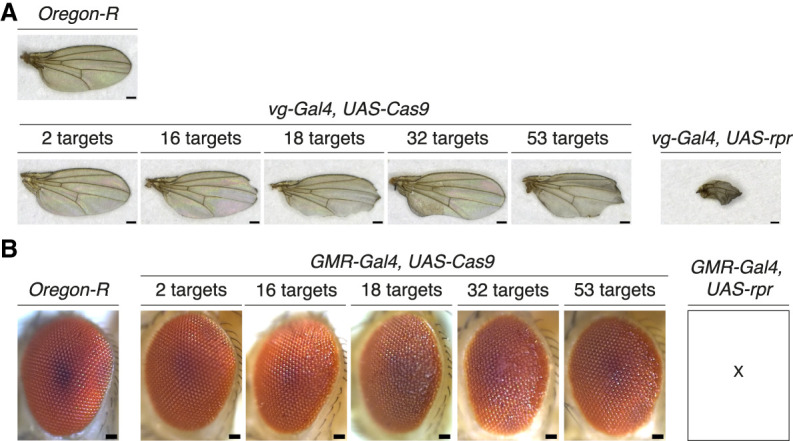
Varying tolerance to DSBs in somatic cellular domains in the wing and eye. (*A*) Wing morphology of flies in which Cas9 was targeted to different numbers of genomic sites (*bottom left* panels) or in which the apoptotic activator *rpr* was expressed (*bottom right* panel) in the *vg-Gal4* domain. (*Top* panel) Wild-type wing morphology is shown (*Oregon-R* strain). (*B*) Eye morphology of flies in which Cas9 was targeted to different numbers of genomic sites or in which the apoptotic activator *rpr* was expressed in the *GMR-Gal4* domain. X indicates no larvae, pupae, or adult progeny were produced. Scale bars: *A*, 100 μm; *B*, 50 μm.

Due to its role in genetic inheritance, the germline is hypothesized to be less tolerant to DNA damage than somatic cells, favoring apoptosis over error-prone DNA repair (Bloom et al. 2019). To examine the sensitivity of somatic cells to DSB dosage, we expressed Cas9 in a mitotic domain in the wing (*vg-Gal4*) and combined this with a subset of our transgenic lines constitutively expressing gRNAs. The *vg* phenotype, which is characterized by reduced wing size and “notched” outer wing margins (Simmonds et al. 1997), was observed in the presence of gRNAs and became more pronounced with increasing numbers of Cas9 targets (Fig. 7A). However, this phenotype was consistently less severe than what we observed when driving expression of the proapoptotic gene *rpr* in the same domain, even when targeting up to 53 sites at once. This indicates that Cas9-induced DSBs did not result in domain-wide cell death.

To examine tolerance to DSBs in postmitotic somatic cells, we expressed Cas9 in the *GMR-Gal4* domain. This domain comprises cells posterior to the morphogenetic furrow that is first established in larval eye discs and mostly contains cells in a postmitotic or differentiating state (Hay et al. 1994; Freeman 1996). Targeting different numbers of loci using a subset of gRNA lines in the GMR domain did not result in a marked reduction in eye size or shape regardless of the number of targets (Fig. 7B). On the other hand, activating apoptosis by expressing *rpr* in the same cellular domain resulted in unviable progeny. These results suggest that postmitotic cells in the eye may be more resilient to DNA damage compared with mitotic cells in the same tissue. Altogether, our findings indicate that responses of different cell types to DNA damage depend on the cellular differentiation state and associated cell division programs, as previously suggested (Fan and Bergmann 2014; Baonza et al. 2022).

## Discussion

TEs successfully proliferate within eukaryotic genomes even though their activity can inflict structural and functional damage on the genome (Rahman et al. 2015; Zhang et al. 2020). Their proliferative success is conditional on their ability to mobilize within the germline. However, compromising the integrity of the germline genome threatens germ cell viability and fertility (Malone et al. 2015). Here, we used *P*–*M* hybrid dysgenesis as a model to study the effects of TE activation in the germline. Against our expectations, single-cell DNA sequencing analysis revealed that during dysgenesis, arrested embryonic PGCs only contain a few new *P*-element insertions despite the accumulation of DSBs. Since a single source of *P*-transposase is sufficient to mobilize multiple *P*-elements in *trans* (O'Hare and Rubin 1983; Engels et al. 1990), the activation of a single full-length *P*-element copy during dysgenesis can in principle mediate the excision of all genomic *P*-elements, including nonautonomous, internally deleted copies. Consequently, DSBs formed during *P*-element excision represent a dominant event, independent of the fact that *P*-elements are only present in the copy derived from the paternal genome during hybrid dysgenesis. Any disruption caused by random insertions would be secondary to the dominant effect of DSBs caused by excisions, as previously hypothesized (Eanes et al. 1988; Eggleston et al. 1988). This is strongly supported by our finding that mimicking DSBs formed during *P*-element excision in the absence of insertions using an engineered Cas9 system is sufficient to trigger germ cell loss during development.

Interestingly, we also observed new insertions in nondysgenic PGCs, where *P*-element activity is largely silenced by the piRNA pathway (Aravin et al. 2007; Brennecke et al. 2008; Teixeira et al. 2017). This observation is in line with lower but nonetheless important levels of *P*-element activity, which relates to the fact that the *P*-element landscape and that of many other TE families are highly dynamic in natural *D. melanogaster* strains (Kofler et al. 2012; Mackay et al. 2012; Rahman et al. 2015). In this context, we propose that DNA damage tolerance thresholds may represent an important mechanism used by germ cells to control TE propagation, acting as a “last line of defense.” Our finding that DSBs at as few as five heterozygous transgenic *P*-elements can elicit nearly complete germ cell loss supports the idea that germ cells are unlikely to tolerate damage from more than a few *P*-elements transposing at a given time. Cells that experience any greater numbers of damage-inducing transposition events are expected to be under strong negative selection.

A further intriguing question is whether DNA damage tolerance of germ cells influences the rate of copy number expansion during *P*-element invasions into naïve strains, which initially lack piRNAs cognate to the invading TE (Bergman et al. 2017; Srivastav and Kelleher 2017; Kelleher et al. 2018; Wang et al. 2023). The severe impact of *P*-element activity on PGC viability and the exclusion of sterile individuals from selection likely present significant hurdles during invasions. Studies of *P*-element invasions in *Drosophila simulans* showed that populations gain ∼0.75 insertions per generation (per haploid genome) but eventually reach a copy number plateau of ∼15 insertions, at which point piRNAs cognate to the *P*-element are abundant (Kofler et al. 2018, 2022). As such, the rate of *P*-element insertions during the initial stages of an invasion seems similar to the rate of new insertions that we observed in dysgenic PGCs, where piRNAs are lacking. Given our results, it would be expected that during the early stages of a TE invasion, when transposition rates and DSB levels are comparatively low, *P*-element activity would be tolerated. However, as *P*-element copy numbers increase within a population, the high sensitivity of PGCs to DSB dosage would lead to an increased frequency of germ cell loss. We speculate that the germline DSB tolerance threshold on the one hand underlies the plateauing in copy number observed in laboratory invasions (Kofler et al. 2018, 2022) and on the other hand creates the selective pressure for the TE silencing machinery against the *P*-element to become activated throughout the population. Related to this, during the hybridization of populations with high *P*-element copy numbers and those without any *P*-elements, invading elements would only be tolerated when they are introduced via the maternal side, together with the protective small RNAs. In the context of DSB tolerance, the genomic *P*-element content may therefore have a major influence on the dynamics of invasions and the TE silencing machinery.

Why PGCs are so sensitive to DSBs remains unclear. DSBs are known to be particularly harmful to cells due to their propensity to cause genomic rearrangements. Moreover, their repair can introduce mutations regardless of which repair pathway is used (Chapman et al. 2012; Krenning et al. 2019). We found that PGCs are sensitive to the DSB levels regardless of whether DSBs are induced at *P*-elements (which are largely located in promoters and introns of genes) or in other intergenic, noncoding regions. One possible explanation for this is that the DNA damage response machinery may not yet be fully matured in PGCs during the early stages of embryonic development. Alternatively, the fact that PGCs are in a state of cell cycle arrest for most of embryogenesis may prevent timely activation of the Chk2-dependent checkpoint and recruitment of the appropriate repair machinery (Su et al. 1998). At early larval stages, when PGCs normally re-enter the cell cycle at the G_2_-to-M-phase transition, the extent of genome damage may exceed the checkpoint's capacity for repair, and despite initially prolonging cell cycle arrest, most cells are lost soon after, during the first instar larval stage. The importance of the checkpoint in PGCs’ response to DNA damage is supported by the finding that *Chk2* mutants suppress some of the germ cell loss in dysgenesis. In the nematode *Caenorhabditis elegans*, PGCs trigger checkpoint-induced apoptosis in response to persistent UV-induced DNA lesions (Ou et al. 2019), suggesting that this checkpoint-mediated response is conserved. Of note, in *Drosophila* larvae, mutations affecting DNA repair and the checkpoint or repair alone result in hypersensitivity to irradiation-induced DNA damage, but mutations affecting only the checkpoint do not (Jaklevic and Su 2004). Therefore, there are likely other, cell cycle-interacting factors that facilitate DNA damage sensing and repair in PGCs. Transcriptome analysis of PGCs may help to obtain an unbiased and holistic view of the molecular pathways that are activated during dysgenesis.

A further open question is whether PGCs are sensitive to different sources of DNA damage. For instance, a direct comparison of the effects of *P*-transposase and Cas9-induced DSBs is precluded by the fact that the in vivo cleavage efficiencies for these two systems are unknown and unlikely to be measurable using currently existing technologies. Also, DNA cleavage by *P*-transposase is known to generate staggered-ended breaks with 17 bp overhangs, while Cas9 induces staggered-ended DNA breaks with 1 to 3 bp overhangs (Beall and Rio 1997; Zuo and Liu 2016; Shou et al. 2018). It is possible that different types of breaks and repair outcomes may trigger different cellular responses. Our molecular analysis of DSB repair products at Cas9 target sites showed that Cas9-induced DSBs in PGCs were most likely repaired by nonhomologous end joining. In contrast, *P*-element excision sites are frequently repaired by homologous recombination (Engels et al. 1990). Previous work has shown that the choice of repair pathway after *P*-element excision depends on the developmental stage (Preston et al. 2006). Whether repair outcomes and cellular responses to DSBs more generally depend on when damage is induced during development remains to be studied.

In contrast to PGCs and mitotically dividing adult germline cysts, germ cells showed remarkable resilience to DSBs at the postmeiotic stage, where DSB repair is essential to resolve the obligatory meiotic crossovers. When DSBs were induced at this stage, we found that ovary morphology and egg laying were unaffected even though oocytes contained strong DSB signal, indicating that oogenesis can be completed despite high levels of genome damage. We hypothesize that this may be related to the fact that germ cells have already been exposed to meiotic DSBs and repair by this stage of oogenesis. Meiotic DSB repair during early to mid-prophase I involves a specific set of factors (Hughes et al. 2018), but when the repair process fails, cells trigger a meiotic checkpoint that involves the canonical ATM pathway factors *mei-41* and *Chk2* (Ghabrial et al. 1998; Abdu et al. 2002). We did not observe the axial patterning defects associated with meiotic checkpoint activation (Abdu et al. 2002; Shim et al. 2014), indicating that DSBs induced after the meiotic DSB repair process may not trigger this checkpoint. Therefore, and despite the fact that the DSBs that we induced resulted in aberrant zygotic development, our findings are in line with a much lower sensitivity to or even an absence of checkpoint-related DNA damage responses in the postmitotic germline.

With respect to TEs, which must mobilize in germ cells to increase in copy number in the heritable genetic material, the comparatively greater tolerance of postmitotic germ cells to genome damage may have implications for proliferation strategies. Despite high levels of DNA damage endured once meiotic DSBs are repaired, oogenesis can be completed, and on rare occasions, embryos develop and hatch in the subsequent generation. The postmitotic domain may therefore provide the most suitable developmental window for TEs to become active and thereby increase their chances of propagating across generations. Importantly, it has been shown that retrotransposon transcripts produced in the nurse cells can be shuttled to the transcriptionally silenced oocyte via microtubules (Van De Bor et al. 2005; Wang et al. 2018). As such, it is possible that TEs use the nurse cell genome as a platform for transposition in the postmitotic germline.

Finally, another open question is whether the shifts in tolerance to DSBs between different stages of germline development is a specific feature of this cell lineage or rather a phenomenon that occurs in other cell lineages during fate specification and differentiation. We observed lower tolerance to equivalent DNA damage levels in mitotic primordial domains in the wing compared with postmitotic domains within the eye. One explanation for this observation is that the primordial cells may not yet be equipped to cope with genome damage to the same extent as fully differentiated cells. In the case of somatic tissues, it is possible that a subset of mitotic cells may trigger cell cycle-regulated checkpoints that lead to cell death, but these may be more effectively replaced by dividing neighboring cells. Further experiments will be needed to determine whether cell cycle state and associated checkpoints are driving the decision of whether to repair or die.

## Materials and methods

### *Drosophila* genetics and husbandry

*D. melanogaster* stocks were maintained on standard cornmeal medium at 18°C. Flies used for genetic crosses were kept on propionic medium supplemented with yeast at 25°C. For genetic crosses, virgin females and males were added to fresh vials and allowed to lay for 2–3 days at 25°C. Chromosomes carrying mutations and transgenes of interest were individually introduced into the *Harwich* background through serial backcrosses. To induce dysgenesis, crosses were established at 29°C. Female hybrids reared in these conditions were fully sterile, and germ cell loss during development has been characterized in detail in female hybrids (Kidwell et al. 1977; Teixeira et al. 2017). To assess F_1_ female ovary morphology, adult female progeny of the appropriate genotype were collected and kept in fresh vials supplemented with yeast for 1–2 days to “fatten” ovaries prior to dissection. To assess the ability of F_1_ females to generate progeny, 2 to 6 day old F_1_ females were collected, individually crossed to two *w*^*1118*^ males, and allowed to lay for 2–3 days. The number of F_2_ progeny (male and female) emerging from 10 individual crosses was assessed 12 days after crosses were set up (Teixeira et al. 2017).

For egg laying and hatching assays, 10 adult females of the tested phenotype were mated with five “wild-type” *w*^*1118*^ males in fresh vials for 2 days before being transferred to standard embryo collection cages for a further 2 days with apple juice/agar plates changed every 24 h. The total number of eggs laid per cage over the next 24 h was counted, and agar plates containing eggs were incubated for a further 24 h at 25°C prior to the total number of hatched eggs being determined. Egg laying and hatching assays were performed in three biological replicates for each genotype and corresponding control.

The *D. melanogaster* stocks used are shown in Table 1.

**Table 1.** D. melanogaster *stocks used in this study*

Genotype	Source
*w[1118];;*	R. Lehmann, Massachusetts Institute of Technology (MIT)
*[Harwich]; [Harwich]; [Harwich]*	Bloomington *Drosophila* Stock Center (BDSC) 4264
*w[1118];P{w[+mC]=UASp-GFP.E2f1.1–230}26, P{w[+mC]=UASp-mRFP1.NLS.CycB.1–266}4/CyO,P{ry[+t7.2]=en1}wg[en11]; MKRS/TM6B, Tb[1]*	BDSC 55110
*w[1118]; P{w[+mC]=Ubi-GFP.E2f1.1–230}19, P{w[+mC]=Ubi-mRFP1.NLS.CycB.1–266}15/CyO,P{ry[+t7.2]=en1}wg[en11]; MKRS/TM6B, Tb[+]*	BDSC 55123
*w[1118]; Kr[If-1]/CyO,P{ry[+t7.2]=en1}wg[en11]; P{w[+mC]=Ubi-GFP.E2f1.1–230}5,P{w[+mC]=Ubi-mRFP1.NLS.CycB.1–266}12/TM6B, Tb[1]*	BDSC 55124
w[*];; P{ry[+t7.2]=neoFRT}82B tefu[atm-3] e[1]/TM6B, Tb[1]	BDSC 8625
*[Harwich]; [Harwich]/CyO; P{ry[+t7.2]=neoFRT}82B tefu[atm-8] e[1]/TM6B*	BDSC 8624 and 4264
*y[1] mei-41[2]/C(1)DX, y[1] f[1];;*	BDSC 4183
*mei-41[D5] f[1];;; sv[spa-pol]*	BDSC 4236
*sn[3] mei-41[D9]/C(1)DX, y[1] f[1];;*	BDSC 4174
*w[1] mei-41[D1]; [Harwich]; [Harwich]*	Kyoto Stock Center 101010 and BDSC 4264
*y[1] w[1]/Dp(1;Y)y[+]; P{w[+mC]=lacW}mei-P22[P22]; sv[spa-pol]*	BDSC 4931
*[Harwich]; [Harwich]/CyO; P{w[+mC]=lacW}mei-P22[P22]/TM6B*	BDSC 4931 and 4264
*y[1] w[67c23]; P{w[+mC]=lacW}mei-W68[k05603] par-1[k05603]/CyO*	BDSC 10574
*[Harwich]; mei-W68[1]/CyO; [Harwich]*	BDSC 4932 and 4264
*y[1] w[1118];; p53[5A-1-4]*	BDSC 6815
*y[1] w[1118];; p53[11-1B-1]*	BDSC 6816
*[Harwich]; [Harwich]/CyO; p53[11-1B-1]/TM6B*	BDSC 6816 and 4264
*;; Df(3L)W4, ru[1] h[1] e[1] ca[1]/TM6B, Tb[1]*	BDSC 2607
*[Harwich]; [Harwich]; st[1] mus304[D1]/TM6B*	BDSC 922 and 4264
*w; grp[fs1]/SM6*	W. Theurkauf, University of Massachusetts
*[Harwich]; P{ry[+t7.2]=PZ}grp[06034] cn[1]/CyO; [Harwich]/TM6B*	BDSC 12219 and 4264
*w; mnk[p6]/CyO, Act-GFP*	T. Schüpbach, Princeton University
*[Harwich]; mnk[p6]/CyO; [Harwich]*	T. Schüpbach (Princeton University) and BDSC 4264
*y w/w; mnk[p6] grp[fs1]/CyO*	W. Theurkauf, University of Massachusetts
*[Harwich]; mnk[p6] grp[fs1]/CyO; [Harwich]*	W. Theurkauf (University of Massachusetts) and BDSC 4264
*w;; P{nos::egfp-moe::nos 3*′*UTR, [w+]}*	R. Lehmann, MIT
*[Harwich]; [Harwich]; P{nos::egfp-moe::nos 3*′*UTR, [w+]}*	R. Lehmann (MIT) and BDSC 4264
*w;; TM2/TM6c, Sb*	Department of Genetics Fly Facility, University of Cambridge
*w- nos-int;; attp2*	S. Bullock, MRC Laboratory of Moleuclar Biology, Cambridge
*y w M(eGFP, vas-int, dmRFP)ZH-2A;; PBac{y[+]-attP-3B}VK00033*	Department of Genetics Fly Facility, University of Cambridge
*w;; PBac{nos-Cas9, U6-chiRNA P-element TIR, w[+]}3B/TM6c, Sb*	This study
*w;; p53R-GFPcyt(GHP150)*	J. Abrams, University of Texas
*P{w[+mC]=hs-bam.O}18d, w[1118];;*	BDSC 24636
*y w hs-flp φC31; S/CyO; L34.2/TM6B*	Department of Genetics Fly Facility, University of Cambridge
w[*]; P{w[+mC]=UAS-2xEGFP}AH2; P{w[+mC]=elav-VP16.AD}G3A1	Department of Genetics Fly Facility, University of Cambridge
P{ry[+t7.2]=hsFLP}22, y[1] w[*];; P{neoFRT}82B P{Ubi-GFP.D}83	R. Lehmann, MIT
*P{UAS-Dcr-2.D}1, w[1118]; nosP-GAL4-NGT40/CyO; P{Ubi-GFP.E2f1.1–230}5 P{Ubi-mRFP1.NLS.CycB.1–266}12/TM6B, Tb1*	BDSC 24646, 25752, and 55124
*DGRP-21*	BDSC 28122
*DGRP-142*	BDSC 28144
*DGRP-153*	BDSC 28146
*DGRP-374*	BDSC 28185
*DGRP-385*	BDSC 28191
*DGRP-392*	BDSC 28194
*DGRP-399*	BDSC 25192
*DGRP-406*	BDSC 29657
*DGRP-765*	BDSC 25204
*DGRP-801*	BDSC 28234
*DGRP-802*	BDSC 28235
*DGRP-832*	BDSC 28245
*DGRP-907*	BDSC 28262
*DGRP-911*	BDSC 28264
y[1] sc[*] v[1] sev[21]; P{y[+t7.7] v[+t1.8]=nanos-Cas9.R}attP2	BDSC 78782
*w;; nosP-Flag-dCas9-HA-NLS-NLS-GFP/ TM6C, Sb*	F.K.T.
*w;; P{y[+t7.7]=U6-chiRNA-2 targets, w[+]}attP2*	This study
*w;; P{y[+t7.7]=U6-chiRNA-4 targets, w[+]}attP2*	This study
*w;; P{y[+t7.7]=U6-chiRNA-6 targets, w[+]}attP2*	This study
*w;; P{y[+t7.7]=U6-chiRNA-7 targets, w[+]}attP2*	This study
*w;; P{y[+t7.7]=U6-chiRNA-8 targets, w[+]}attP2*	This study
*w;; P{y[+t7.7]=U6-chiRNA-11 targets_1, w[+]}attP2*	This study
*w;; P{y[+t7.7]=U6-chiRNA-11 targets_2, w[+]}attP2*	This study
*w;; P{y[+t7.7]=U6-chiRNA-16 targets, w[+]}attP2*	This study
*w;; P{y[+t7.7]=U6-chiRNA-18 targets, w[+]}attP2*	This study
*w;; P{y[+t7.7]=U6-chiRNA-25 targets, w[+]}attP2*	This study
*w;;P{y[+t7.7]=U6-chiRNA-32 targets, w[+]}attP2*	This study
*w;; P{y[+t7.7]=U6-chiRNA-53 targets, w[+]}attP2*	This study
*w;; P{y[+t7.7]=U6-chiRNA-RpS6, w[+]}attP2/TM6c*	This study
*w;; P{y[+t7.7]=U6-chiRNA-RpL5, w[+]}attP2/TM6c*	This study
*w;; P{y[+t7.7]=U6-chiRNA-RpL8, w[+]}attP2/TM6c*	This study
*w; P{w[+mC]=mat*α*-GAL4-VP16}V2H, P{w[+mC]=osk-GAL4::VP16}A11/CyO*	Vienna *Drosophila* Resource Center (VDRC) 314033
*Sp/CyO; P{bam-Gal4:VP16}/TM6B*	E. Bach, New York University
P{ry[+t7.2]=hsFLP}12, y[1] w[*]; P{Gal4}vg-Gal4 P{y[+t7.7] w[+mC]=UAS-uMCas9}attP40	VDRC 340011
P{ry[+t7.2]=hsFLP}12, y[1] w[*]; P{Gal4}GMR-Gal4 P{y[+t7.7] w[+mC]=UAS-uMCas9}attP40	VDRC 340012
P{ry[+t7.2]=hsFLP}12, y[1] w[*]; P{y[+t7.7] w[+mC]=UAS-Cas9.P2}attP40	BDSC 58985
P{hsFLP}12, y1 w*; P{UAS-Cas9.P2}attP2/TM6B, Tb1	BDSC 58986
*w[1118];; P{w[+mC]=UAS-rpr.C}27*	BDSC 8523
*Oregon-R-C*	BDSC 5

### Immunofluorescence and confocal microscopy

Embryos (0–14 h old) were collected on standard apple agar plates supplemented with yeast paste. Embryos were dechorionated in 2.5% bleach for 2 min and subsequently incubated in fixation solution containing 9% formaldehyde in 1× PBS and heptane (1:5) for 30 min (Seifert and Lehmann 2012). Fixed embryos were hand-devitellinized in PBT (1× PBS, 0.1% Triton X-100) and blocked in PBTB (1× PBS, 0.2% Triton X-100, 1% bovine serum albumin) for 1 h. Larvae were collected at 24–48 h (first instar) and 72–120 h (third instar) and dissected in ice-cold 1× PBS (Teixeira et al. 2017). Larval tissue was fixed in 4% formaldehyde for 20 min, washed three times in PBT (1× PBS, 1% Triton X-100), and blocked in PBTB for 1 h (Maimon and Gilboa 2011). Adult ovaries were dissected in cold 1× PBS and fixed in 4% formaldehyde for 20 min. Ovaries were washed three times in PBTB for 20 min each and blocked in PBTB for 1 h. Blocked samples were incubated in primary antibodies diluted in PBTB overnight at 4°C. Samples were washed three times in PBTB and then incubated in secondary antibodies diluted in PBTB for 2 h at room temperature in the dark. Samples were washed three times in PBTB and mounted in VectaShield medium containing DAPI (Vector Laboratories). Fluorescent images were acquired on a Leica SP8 confocal microscope using 20× dry or 40× oil objectives.

To quantitate fluorescent signal, *Z*-stacked images of embryonic and larval gonads were acquired. Female embryonic gonads were identified by the presence of PGCs and the absence of Vasa-positive male-specific somatic gonadal precursor cells (Renault 2012). To quantify fluorescent signal in embryonic and larval PGCs, images were loaded into Fiji ImageJ (Schindelin et al. 2012). Boundaries of PGC and epidermal cell nuclei were defined as regions of interest (ROIs). For each ROI, area, mean intensity, and area-integrated density were measured on GFP and RFP channels. To account for background signal, measurements were also taken on nonepidermal somatic cells (RFP- and GFP-negative) within the same image slices. For each PGC and epidermal cell ROI, the corrected total cell fluorescence (CTCF) was calculated as previously described (McCloy et al. 2014).

A Leica epifluorescence microscope fitted with an LED light source and 488 nm filter was used to quantify pH2Av-positive oocytes. The number of ovarioles containing at least one stage 2–6 egg chamber with pH2Av signal was counted manually across two biological replicates.

The antibodies used were mouse anti-1B1 (1:200; Developmental Studies Hybridoma Bank [DSHB]), rabbit anti-Vasa (1:5000; R. Lehmann, Massachusetts Institute of Technology [MIT]), rat anti-Vasa (1:50; DSHB), mouse anti-pH2Av (1:200; DSHB UNc93-5.2.1-s), rat anti-RFP (1:500; Chromotek RMA5F8), chicken anti-GFP (1:500; Aves Labs GFP-1010), mouse anti-α-spectrin (1:200; DSHB 3A9 323/M10-2), rabbit anti-Cas9 (1:1000; Diagenode C15310258-100), Alexa Fluor Plus 488 goat antimouse (1:250; Invitrogen A32723), Alexa Fluor 647-AffiniPure donkey antirabbit (1:250; Stratech 711-605-152-JIR), Alexa Fluor 488 goat antichicken (1:250; Invitrogen A11039), Alexa Fluor 594 donkey antirat (1:250; Invitrogen A21209), and Cy3-AffiniPure donkey antimouse (1:250; Stratech 715-165-150-JIR).

### Isolation of PGCs from embryos

Reciprocal dysgenic and nondysgenic crosses between the *Harwich* and *w*^*1118*^ strains expressing the fluorescent PGC marker transgene *P(nos::egfp-moe::nos-3*′*UTR[w+])* were established in separate cages (∼250 flies per cage) fitted with apple agar plates supplemented with yeast paste for 2 days at 25°C (Sano et al. 2005; Teixeira et al. 2017). In parallel, cages of the *w*^*1118*^ stock (lacking the germ cell marker) were established as a negative control. Cages were moved to 29°C to induce dysgenesis for 1 day prior to collection. Embryos were collected for 4 h and aged at 29°C. Eleven hour to 16 h old embryos were dechorionated in 2.5% bleach for 2 min, washed with water, and incubated in cell-sorting buffer (“balanced saline”) for 2 min (Chan and Gehring 1971). Embryos were manually dissociated by Dounce homogenization in 5 mL of cold cell-sorting buffer. The homogenate was sequentially filtered through 100 and 20 μm cell strainers (Celltrics). Live/dead staining with Zombie Aqua fluorescent dye (BioLegend) was used to exclude dead cells. The dye was reconstituted in DMSO according to the manufacturer's instructions and diluted 1:100 in PBS. One milliliter of cell homogenate was transferred to separate microcentrifuge tubes as controls for live/dead staining. Cells were pelleted, washed, resuspended in cold 1× PBS (unstained controls) or dye solution, and incubated for 10 min at room temperature. Cells were washed twice and resuspended in cold cell-sorting buffer. Cells were sorted on a BD FACS Aria Fusion cytometer at the National Institute for Health and Care Research Cambridge Biomedical Research Centre Cell Phenotyping Hub Facility. Lasers (488 and 568 nm) were used to identify the GFP-positive, Zombie Aqua-negative cell population. Single live, GFP-positive cells were sorted into REPLI-g cell storage buffer (Qiagen).

### DNA extraction and amplification

Genomic DNA from freshly sorted single cells was extracted and amplified using the REPLI-g Advanced Single Cell kit (Qiagen) according to the manufacturer's instructions. Whole-genome amplified DNA was quantified using the Qubit dsDNA broad range assay kit (Invitrogen). Size distribution of amplicons was determined using the TapeStation genomic DNA screen tape assay (Agilent).

Bulk genomic DNA was extracted from 10 nonvirgin female adult flies using the Quick-DNA micropreparation kit (Zymo) and quantified by Qubit. Bulk-extracted DNA was not amplified prior to library preparation.

### Quantitative PCR screen to determine cell sex

To distinguish male (XY) and female (XX) sorted PGCs, a quantitative PCR (qPCR) screen was set up based on the presence or absence of the Y chromosome. Sets of primers corresponding to the CDSs of Y-chromosome genes *ARY* and *FDY* were designed using FlyBase and primer3. Sets of primers corresponding to genes on the autosomes (*Dmn*, *Und*, and *nos*) were used as copy number controls. qPCR reactions were set up in technical duplicates using LightCycler 480 SYBR Green I master mix (Roche), 5 ng of amplified DNA, and 1 μM forward and reverse primers and run on a LightCycler 480 machine (Roche) using the following protocol: 10 min at 95°C, 10 sec at 95°C, and 1 min at 60°C for 45 cycles. Melting curves were used to verify primer specificity. Female cells were identified by the lack or low amplification of Y-chromosome genes (high *C*_*T*_ values and unspecific priming on the melting curve) compared with autosomal genes.

The oligonucleotides used were ARY_F (AGATACTTGGCGAGCAATGG), ARY_R (AGCGGCAATAATCAACCAAG), FDY_F (AACCAGGGCAGGTTCAACAA), FDY_R (ACGGAGCAAACACGAGAACA), Dmn_F (AGACGCCTGGAAGTAAGCAG), Dmn_R (GTAAGGCGGCTCAACTTGTC), Und_F (GCAAGAAAAGCGGTCAGACT), Und_R (CGTGTTGATACGGTCCAGAG), nos_F (ATCTCGGTCGCATGTCCTAC), and nos_R (CAGATGCTCCCGGTAGTTGT).

### Short read DNA sequencing

DNA sequencing libraries were prepared using the DNA preparation kit (Illumina) with 500 ng of whole-genome amplified DNA (single PGC samples) or bulk DNA (whole fly, no amplification) as input. Libraries were multiplexed using Nextera index adapter oligos (Illumina) and quantified by Qubit (Invitrogen). Library size distribution was determined by Bioanalyzer (Agilent). Multiplexed libraries were sequenced on a NovaSeq 6000 system (Illumina) as paired-end, 150 nt reads.

Reads were mapped to the reference genome dm6 using BWA MEM (Li and Durbin 2009). Alignments were filtered for unique mappers, and a map quality cutoff of 30 was applied using Samtools (Li et al. 2009). The total number of mapped reads and the mapped read count per chromosome were obtained using the Samtools utility idxstats. Overall genome coverage was calculated by multiplying the total mapped read count by paired-end read length (2 nt × 150 nt) and dividing by the dm6 reference genome size. To obtain read coverage over 10 kb windows, dm6 was indexed and sorted using Samtools and the command line utility sort. Bedtools was used to define coordinates of 10 kb windows (autosomes and X chromosome) and determine the number of reads per window for each sample (Quinlan and Hall 2010). Coverage per window was determined by multiplying the read count per window by paired-end read length and dividing by window size. The (*k*,*e*)-mappability of the reference genome dm6 was obtained using genmap, with a k-mer size of 150 and mismatch number of 2 (Pockrandt et al. 2020). To obtain the average mappability per 10 kb window, genome-wide mappability scores of 150 k-mers for each 10 kb window were determined using the Bedtools tool map with the option mean.

### Long read DNA sequencing

High-molecular-weight (HMW) genomic DNA was extracted using the Genomic Tips 100/G kit (Qiagen) following a modified version of the manufacturer's instructions. For each genotype of interest, 60 female flies were flash-frozen in liquid nitrogen, homogenized in lysis buffer, vortexed, and incubated for 1 h at 37°C. Following incubation with Proteinase K, samples were centrifuged at 5000*g* for 10 min at 4°C. After the addition of isopropanol, DNA eluate was centrifuged at 10,000*g* for 30 min at 4°C. Purified DNA was resuspended in elution buffer (Qiagen) and dissolved for 1 h at 37°C and for >12 h at 4°C. Concentration and purity (A_260_:A_280_) were determined by Qubit and NanoDrop (Thermo Fisher). DNA size range was determined using TapeStation (Agilent).

Libraries for long read sequencing were prepared using 3–7 μg of HMW DNA as input for the Oxford Nanopore Technologies (ONT) ligation sequencing kit (SQK-LSK110) according to the manufacturer's instructions with the following modifications. DNA repair and end-prepared reactions were incubated for 30 min at 20°C and for 30 min at 65°C. Samples were diluted in 90 μL of elution buffer (NEB), mixed with 120 μL of AMPure XP beads (Beckman Coulter), and incubated on a rotator mixer for 10 min at room temperature. Bead-bound DNA was washed twice with 300 μL of 80% ethanol. Beads were resuspended in 63 μL of elution buffer (NEB) and incubated for 30 min at 34°C prior to sample elution. End-prepared DNA was quantified using the Qubit dsDNA broad range kit. Sequencing adapter ligation reactions were incubated for 1 h at room temperature. Libraries were subsequently diluted in 50 μL of elution buffer for bead cleanup as described above. Beads were resuspended in 26 μL of elution buffer (ONT) and incubated for 30 min at 34°C prior to sample elution. Final libraries were quantified by Qubit.

Libraries were sequenced on SpotON flow cells (version R9, FLO-MIN106D) primed using the flow cell priming kit (EXP-FLP002) on a MinION Mk1C sequencing device (ONT) according to the manufacturer's instructions. To generate sufficient genome coverage, two genotypes were run on each flow cell for ∼24 h each. Flow cells were washed between runs using the flow cell wash kit (EXP-WSH004).

### Genome assembly

ONT reads that passed quality control (*Q*-score ≥8) were used as input for the Flye genome assembler (Kolmogorov et al. 2019). ONT reads were then used to polish the Flye output draft assembly using the Medaka sequence correction tool (ONT) and scaffolded on the dm6 reference genome using the D-GENIES alignment tool (Cabanettes and Klopp 2018). Assemblies were further polished using Illumina short read data for three consecutive runs of the Racon polishing tool (Vaser et al. 2017).

### Transposon and *P*-element insertion analysis

To identify existing *P*-element insertions in the *Harwich* strain used for hybrid dysgenesis crosses, the search_repeat_copies Perl script was run with the *P*-element consensus sequence (FlyBase) and genome assembly as inputs (Gebert et al. 2021). In parallel, TEMP was run on Illumina data from the *Harwich* strain and sorted PGCs (Zhuang et al. 2014). Insertions identified by TEMP and supported by fewer than two reads were removed from analyses. To validate the presence and determine the precise coordinates of *P*-element insertions relative to dm6, short read alignments generated with BWA MEM and long read alignments generated with minimap2 (Li 2018) were loaded into the Integrative Genomics Viewer (IGV) (Robinson et al. 2011). Soft-clipped reads overlapping the insertion site were extracted, and insertion coordinates were determined by BLAST against the *P*-element consensus sequence and dm6 (FlyBase). Coverage frequencies for insertions with ≥10 supporting reads were obtained from the variant support values output by TEMP.

Total genomic TE copy numbers were determined by mapping reads to the complete set of *D. melanogaster* TE consensus sequences (FlyBase) using BWA MEM and Samtools with map quality ≥30. To normalize read counts for each TE family by genome coverage, read counts were multiplied by the paired-end read length and divided by TE consensus length and genome coverage (Gebert et al. 2021). Mean normalized TE counts in dysgenic and nondysgenic female and male PGC genomes were determined.

### DNA qPCR for copy number validation

Genomic DNA was extracted from individual female adult flies from stocks of interest using QuickExtract solution (Lucigen) according to the manufacturer's protocol. Primer sets for the *P*-element 5′ and 3′ TIRs were designed using the *P*-element consensus sequence (FlyBase) and primer3. For qPCR reactions, 1 μL of DNA was added to a master mix containing 2× LightCycler 480 SYBR Green I master mix, 1 μM forward and reverse primers, and nuclease-free water. For each genotype and primer set, reactions were performed in two technical replicates. Autosomal genes *Dmn*, *nos*, and *Und* were used to benchmark copy number. qPCR reactions were run as described above. Threshold cycle (*C*_*T*_) values were averaged across replicates. For each sample, *C*_*T*_ values for the two positive control genes were averaged. For each *P*-element region, *C*_*T*_ values were subtracted from the average autosomal gene *C*_*T*_ value (Δ*C*_*T*_ autosomal gene − Δ*C*_*T*_ *P*-element). Copy number estimates were calculated as $2^{-(\Delta C_{T}\;\mathrm{autosomal}\;\mathrm{gene}\;-\;\Delta C_{T}P\text{-}\mathrm{element})}$.

The oligonucleotides used were P_5′_F (GTGGTCCCGTCGAAAGCC), P_5′_R (AAATTCGTCCGCACACAACC), P_3′_F (CCACGGACATGCTAAGGGTT), P_3′_R (TCGGCAAGAGACATCCACTT), P_internal_F (CGTGCCGAAGTGTGCTATTA), and P_internal_R (TGTCTGACCTTTTGCAGGTG).

### CRISPR–Cas9 target sequence selection

A gRNA sequence containing a protospacer-adjacent motif (PAM) was identified within the *P*-element TIRs (1–20 bp and 2888–2907 bp of the full-length *P*-element sequence) using the *P*-element consensus sequence (FlyBase) and published gRNA design guidelines (Port et al. 2014).

We established a Perl-based program (GenoScythe) that performed a heuristic search for gRNA sequences with different numbers of genomic targets within dm6. Genomic positions for introns and TEs were extracted from GTF and RepeatMasker files with annotations based on dm6 (FlyBase). An initial list of 500,000 sequences was generated by extracting 20 nt short sequences from random positions within genomic intron and TE regions containing a 5′ G and followed by a PAM according to published gRNA design guidelines (Port et al. 2014). Sequences with stretches of at least four simple repeats (e.g., AAAA or ACACACAC) were excluded. The number of sequences originating from each chromosome was proportionate to the corresponding chromosome length. Unique sequences were given identifiers and mapped to the reference genome using Bowtie (Langmead et al. 2009). Genomic hits were then counted for alignments with a perfect match in the 12 nucleotides adjacent to the PAM (“proximal” part) and with a maximum of two mismatches in the eight nucleotides furthest from the PAM (“distal” part). Sequences with off-target hits (e.g., outside introns and TEs) and sequences with hits that had mismatches were filtered out at this stage. Similarly, sequences with any hits outside of the specified set of chromosomes were discarded. The final output tables contained the sequence, total number of hits, number of hits on each chromosome arm, hit coordinates, and strand.

The presence of the gRNA sequences targeting multiple genomic sites was validated within the genomes of the *nos-int;attP2* and *w;TM2/TM6* strains used to generate balanced gRNA-expressing lines and the *nos-Cas9* strain. Briefly, for each strain, short read (Illumina) and long read (ONT) DNA sequencing data were obtained as described above. Genomes were assembled and polished as described above. The set of dm6-derived target sequences was mapped to each genome assembly using Bowtie. The resulting SAM files contained the number of hits, location, and strand (+ or −) for each target sequence within each assembly. The number of diploid target sites was determined as double the average haploid number of target sites across the three genomes.

RP genes with an associated *Minute* (haploinsufficiency) phenotype were identified previously (Marygold et al. 2007). CRISPR optimal target finder was used to identify Cas9 target sequences within the CDS for each gene (FlyBase, https://flybase.org; Gratz et al. 2014).

gRNA target sequences (including PAMs) were *P*-element TIR (GCATGATGAAATAACATAAGG_TGG), 2-targets (GATTAATAGCCTAAACTGTC_CGG), 4-targets (GCATCCTCGGTTTTACCTAT_CGG), 6-targets (GGGTTCCTATAGCAGCTGAA_GGG), 7-targets (GTGAGCATGCGTCCGAATCG_AGG), 8-targets (GGCACTAGTAACCAAACTAG_AGG), 11-targets (1) (GTGCTGTTCTCTGCTCTGGC_GGG), 11-targets (2) (GAGGCCTCCACAACTATGTC_TGG), 16-targets (GAATGTGGCTCTCGGTGATT_CGG), 18-targets (GACAGGAAGGAAAGTAGGGG_AGG), 25-targets (GAGCCTAGCCGCGGCTCCCT_CGG), 32-targets (GTCTTTAATATGTTGAGCAG_TGG), 53-targets (GGGGTGAGGATAGGTAATGG_GGG), RpS6 (GAAGCGTATGGGACAGGTTG_TGG), RpL5 (GGTACCAAGTCAAGTTCCGA_AGG), and RpL8 (GGGAGCTGGTTCCGTGTTCA_AGG).

### CRISPR–Cas9 target sequence cloning and transgenesis

The *TIR-gRNA* sequence was cloned into the pU6-BbsI-chiRNA plasmid (Addgene 45946) using the FlyCRISPR protocol (Port et al. 2014). The resulting pU6-chiRNA cassette was amplified by PCR and inserted into the pnos-Cas9-nos plasmid (Addgene 62208) at the NheI restriction site. gRNA sequences targeting different numbers of genomic sites and RP-gRNA sequences were individually cloned into the pU6-BbsI-chiRNA plasmid. gRNA cassettes were amplified by PCR and individually inserted into a pWalium22 backbone containing a *mini-white* selection marker and *att*B site (*Drosophila* Genomics Resource Center 1473). Final constructs were assembled by Gibson assembly (NEB) according to the manufacturer's protocol. Plasmid assembly was confirmed by Sanger sequencing and restriction digestion analysis. Plasmid DNA was injected into embryos from the *vas-int;attP-3B* stock (for transgenesis of the TIR-gRNA, nos-Cas9 construct) and from the *nos-int;attP2* stock (for transgenesis of the multitarget gRNA and RP-gRNA constructs) at the Department of Genetics Fly Facility at the University of Cambridge. Final, balanced transgenic lines were generated by backcrosses to *w;TM2/TM6* flies.

### CRISPR–Cas9 molecular validation

For molecular analysis of DSB repair products at Cas9 target sites, PCR primers were designed to amplify ∼250 to 2200 bp regions containing the target sequences and flanking regions for “hit” locations present in all of the *nos-Cas9*, *nos-int;attP2*, and *w;TM2/TM6* genome assemblies (primer3). To validate PCR primer specificity and efficiency, bulk-extracted genomic DNA from these strains was used to set up PCR reactions for each primer set using quick-load Taq 2X master mix (NEB) according to the manufacturer's protocol. Amplicon size was validated by gel electrophoresis. Amplified DNA was purified using the QIAquick PCR purification kit (Qiagen) prior to Sanger sequencing analysis. To evaluate Cas9 editing efficiency, F_1_ virgin females from crosses between the *nos-Cas9* line and gRNA lines with two to 11 target sites (below the full germ cell loss-inducing threshold) were crossed to *w*^*1118*^ males. F_2_ adult females were collected for DNA extraction using QuickExtract solution, and PCR amplification and Sanger sequencing analysis were performed as described above on ≥10 biological replicates per target site. Sequences perfectly matching the wild-type strains were classified as unedited, while sequences containing indels and mutations around the Cas9 target site were classified as edited.

The oligonucleotides used for validation were 2_targets_2L_F (GGCTGCGAGACCTGACAATT), 2_targets_2L_R (AAATGTGTGGGCGTGGAAAA), 4_targets_3L_F (TTGATTCACGGTCCGCACAT), 4_targets_3L_R (GCTATTCCGTGGTGTGTGGA), 4_targets_2R_F (CCGCTCACACCAATTCACCA), 4_targets_2R_R (TTCAGCCGTACCAATGCACA), 6_targets_2L_F (CACACAACGTCGTTTGGTGT), 6_targets_2L_R (ACTTAGTTGCCTTGCCCTTCA), 6_targets_2R_F (TGCCTTGGCTTGTTCTGCTA), 6_targets_2R_R (TCGCCAGACTAACGTGCTAG), 6_targets_3L_F (AAGCGACAACAACGGCTTTC), 6_targets_3L_R (CACCACCAGTCTGAGTTGCT), 7_targets_3R_F (GCATGTCTTCTCCGATCGCT), 7_targets_3R_R (ACGCTATTTTGCCGTTACCG), 7_targets_3L_F (ACAAGGTGTTAGTTCATGGGCA), 7_targets_3L_R (AGAAATGACCTCTTGCGGCA), 7_targets_2R_F (CGTTACTGAATGGAAGCGGT), 7_targets_2R_R (TCGTCGATGTTCCTGGCAAT), 8_targets_2R_F (AGAATGGAGTGCGCTTATGC), 8_targets_2R_R (TCATGTGACTGCTCTTGGGT), 8_targets_3L_239_F (CGTGATGTGGGATCAACGTC), 8_targets_3L_239_R (AAAACTCCACCTCTCCGTCC), 8_targets_X_2181_F (GAAAATACTAGGGGCACGTCC), 8_targets_X_2181_R (CGCTTTTGTGACCGGGTTAA), 11_targets_1_2L_F (ATCAACCCTTCTCCACCCTG), 11_targets_1_2L_R (GTGCAGTCTGTTAGTGGTGC), 11_targets_1_2R_208_F (TTAATCCCTGGCTGGTTCGT), 11_targets_1_2R_208_R (TACTGTGGCATCGATGTGGT), 11_targets_1_3R_582_F (ATGAAGGTGGAGGTCAGGTG), 11_targets_1_3R_582_R (CGGTGTACTGCCCTCCTAAT), 11_targets_1_3R_899_F (GAGTGGAGTGGCTCGAAAGG), 11_targets_1_3R_899_R (GGATTTATACCCCGCGCAAC), 11_targets_1_3R_349_F (GCAAGAGGACCACATCAACC), and 11_targets_1_3R_349_R (AACAGCAGGGGTAGATGGTC).

### Statistical analysis

All experiments were conducted at least three independent times. Statistical analysis was performed using GraphPad Prism software. Statistical significance was tested by unpaired *t*-test, one-way ANOVA, and Tukey's multiple comparisons test. Pearson correlation coefficient was determined with 95% confidence intervals. No statistical methods were used to predetermine sample size. Experiments were not intentionally randomized or intentionally ordered. Investigators were not blinded to allocation during experiments and outcome assessment.

### Resource availability

Further information and requests for resources or reagents should be directed to the lead contact, F.K.T. (fk319@cam.ac.uk).

### Materials availability

Transgenic *Drosophila* lines generated in this study are available on request.

### Data and code availability

DNA sequencing data and genome assemblies generated in this study have been deposited at the NCBI Sequence Read Archive (SRA; https://www.ncbi.nlm.nih.gov) under project number PRJNA1044074. Custom code used in this study has been deposited at Github (https://github.com/d-gebert/GenoScythe). Any additional information required to reanalyze the data reported in this study is available on request.

## Supplementary Material

Supplement 1

Supplement 2

Supplement 3

Supplement 4

Supplement 5
