# Supplementary material for: Tolerance thresholds underlie responses to DNA damage during germline development
Source: Genes Dev. 2024 Jul 1;38(13-14):631–54. doi: 10.1101/gad.351701.124 (PMC11368186; doi:10.1101/gad.351701.124)
Supplement: Supplement 5 [file Supplemental_Data.pdf]

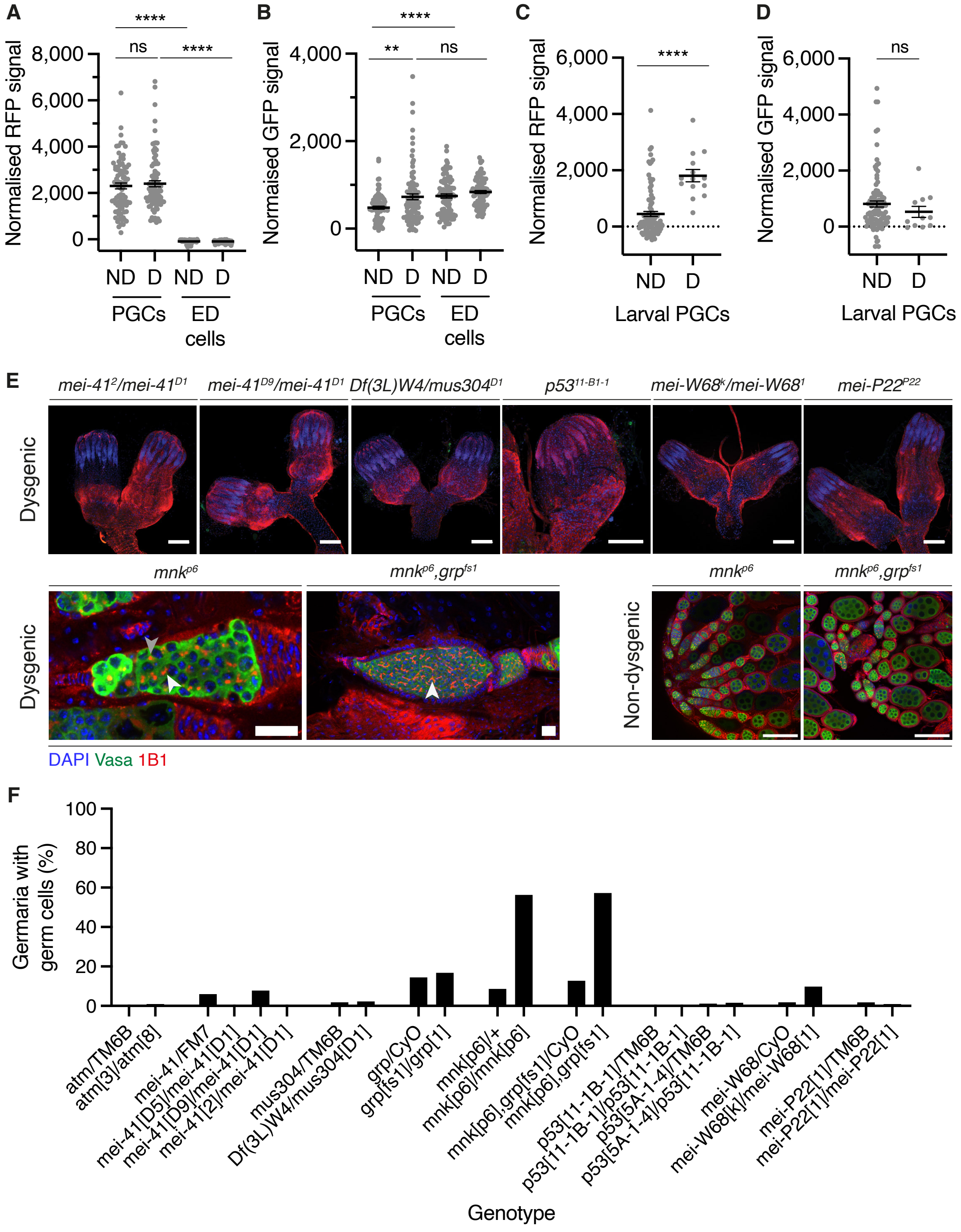

Supplemental Figure S1. Germ cell loss is suppressed in dysgenic progeny mutant for *Chk2/mnk*. A-D. Quantitation of RFP (RFP::CycB; S/G2-phase) and GFP (GFP::E2f1; M/G1/G2-phase) fluorescent signal in embryonic PGCs and epidermal (ED) cells (A and B) and first instar larval PGCs (C and D) shown in Fig. 1E, normalised by signal in somatic epithelial (non-ED) cells. Each data point represents one PGC or ED cell in dysgenic (D) or non-dysgenic (ND) progeny. Black line is mean, error bars are ± SEM. **** $p \leq 0.0001$, ** $p \leq 0.01$, * $p < 0.05$ and ns $p > 0.05$, unpaired *t*-tests. Scale bars, 20 µm. **E.** Adult ovaries from dysgenic hybrids carrying mutations in *mei-41*, *mus304*, *p53*, *mei-W68*,

mei-P22, mnk or *mnk, grp* (in a homozygous or transheterozygous state), labelled with DAPI (blue), Vasa (green) and 1B1 (red). Ovaries from non-dysgenic hybrids mutant for *mnk* or *mnk, grp* are shown. Grey and white arrowheads indicate dot-like spectrosomes and branched fusomes, respectively. Scale bars, 100 µm (top row and bottom right panels) or 20 µm (bottom left panels). **F.** Number of germaria with germ cells from ovaries shown in (E) and Fig. 1F. n > 64 germaria per genotype.

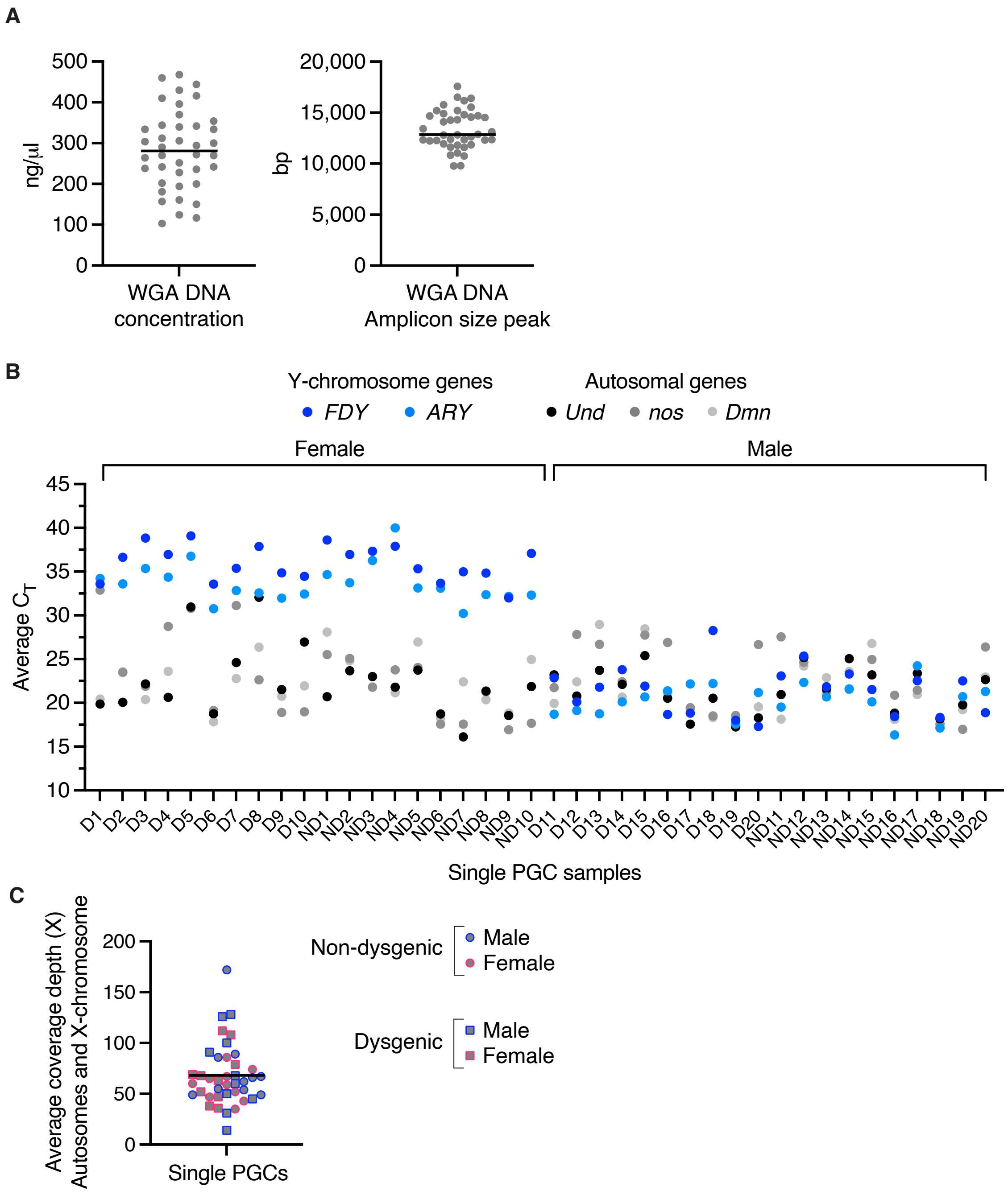

Supplemental Figure S2. WGA DNA from single sorted PGCs generates high overall genome coverage in DNA-sequencing data and can be used to determine cell sex. A. Concentration (left panel) and amplicon size peak (right panel) of WGA DNA from sorted PGCs. Each data point represents one PGC sample. Black lines indicate mean. **B.** Average threshold amplification cycle (C_T) values for Y-chromosome genes *FDY* (dark blue) and *ARY* (light blue) and autosomal genes *Und* (black), *nos* (dark grey) and *Dmn* (light grey) for the 40 PGC samples, as determined by qPCR on WGA DNA. High C_T values (≥30) for Y-chromosome genes designate female PGCs, low C_T values (<30) designate male PGCs. **C.** Average coverage depth across the autosomes and X-chromosome in 40 whole-genome

PGC data sets. Dysgenic (squares) and non-dysgenic (circles), male (blue) and female (magenta) PGCs are indicated. Black line represents mean.

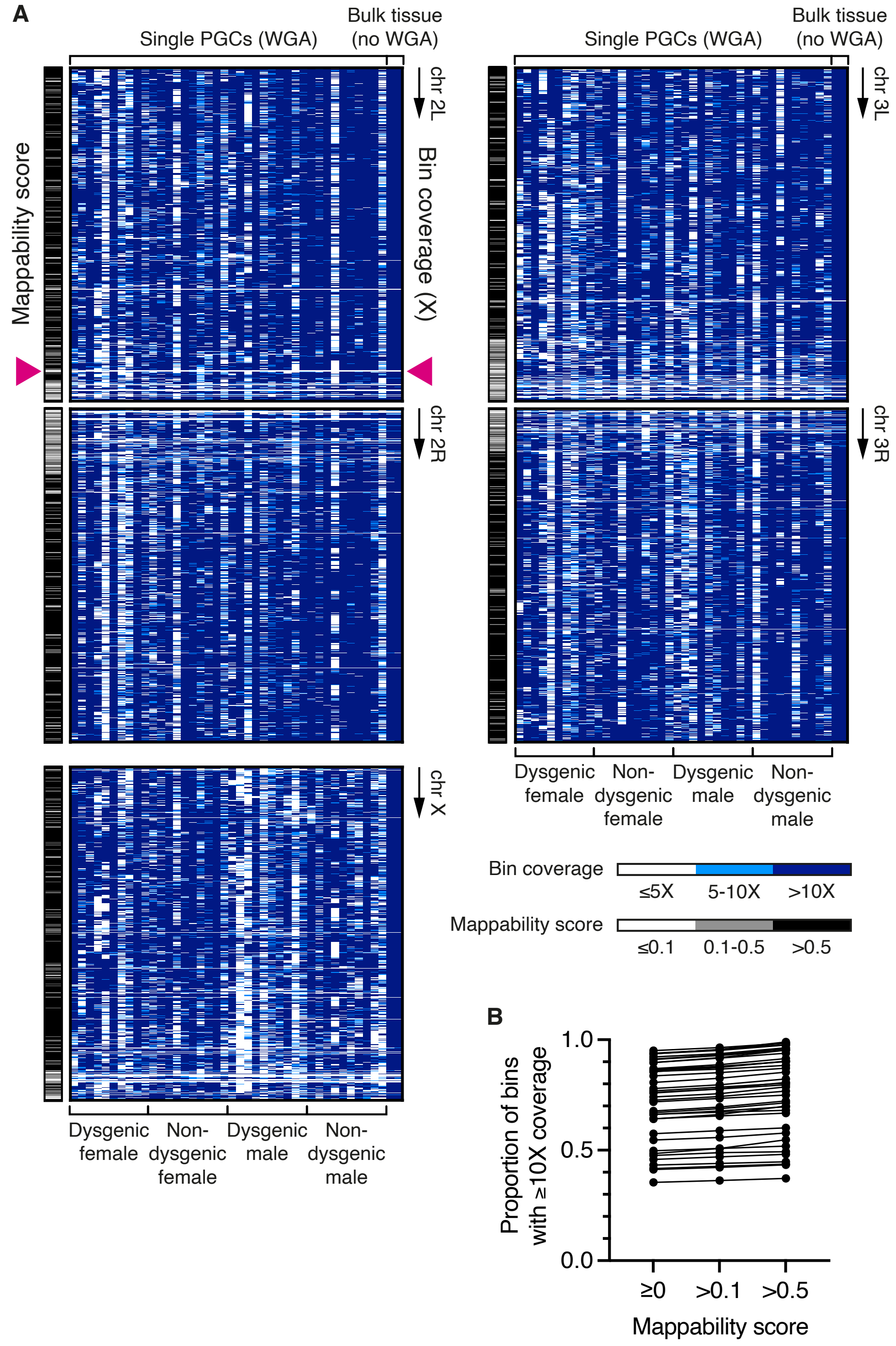

Supplemental Figure S3. Whole-genome sequencing of WGA DNA from single PGCs produces variable genome coverage. A. Heatmaps showing coverage depth (≤5X, white; 5-10X, light blue; >10X, dark blue) over 10-kb genomic regions along chromosome arms 2L, 2R, 3L and 3R and the X-chromosome (rows) for 40 PGCs and 2 non-WGA (bulk tissue) samples (columns). Mappability scores (<0.1, white; 0.1-0.5, grey; >0.5, black) for 10-kb windows in the *dm6* reference genome are shown to the left of each coverage heatmap. Magenta arrowheads indicate a region with low mappability and low coverage depth in all samples, as one of few exceptions to the overall random distribution of coverage

variability in PGC data sets. **B.** Proportion of 10-kb windows with ≥10X read coverage when 3 different minimum mappability scores are applied. Data points represent individual PGC genomes.

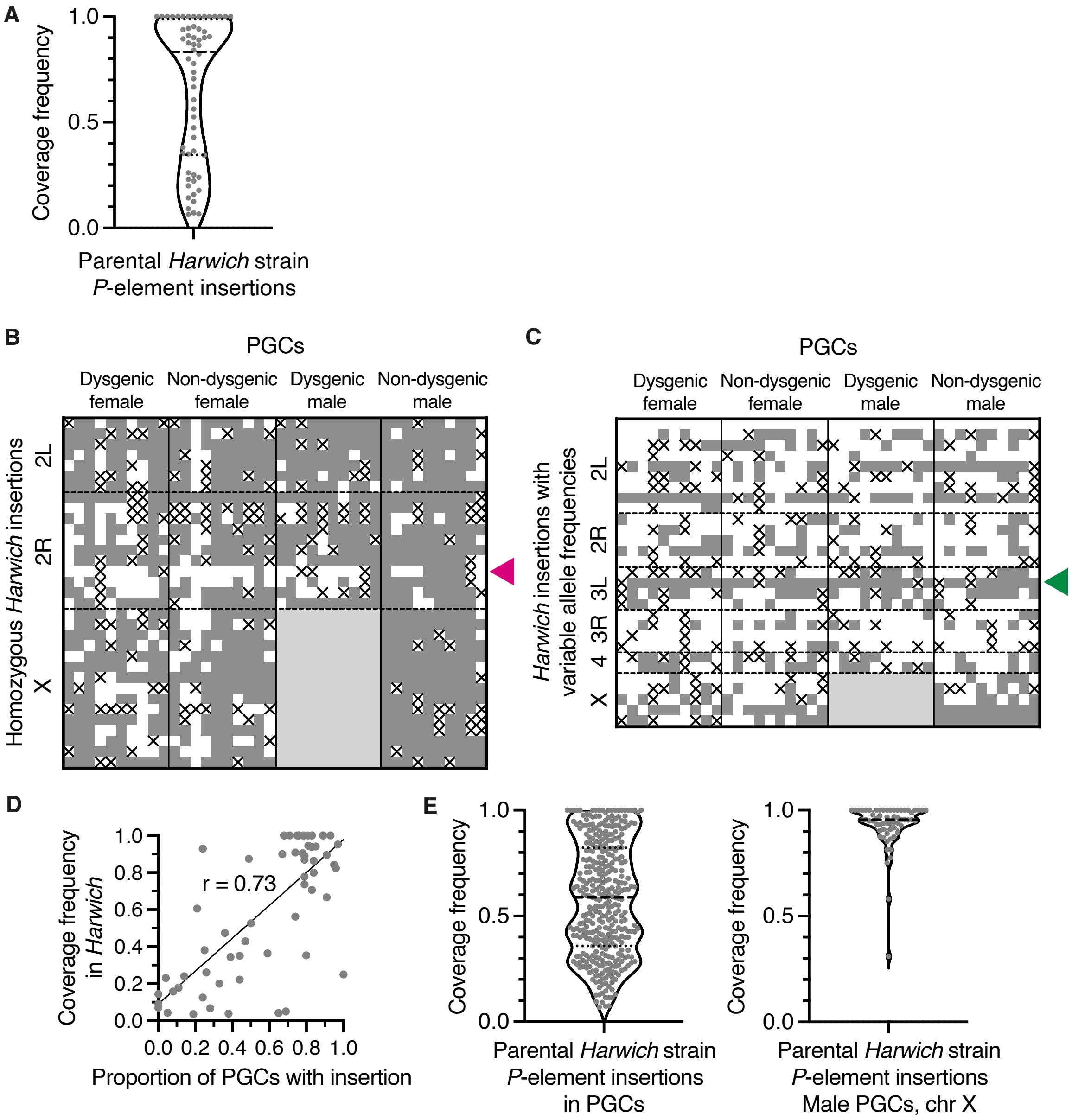

Supplemental Figure S4. High detection rate of vertically transmitted *P*-elements in PGCs. A. Coverage frequencies representing zygosity of *P*-element insertions (grey data points) in the *Harwich* genome. Based on a minimum allele frequency of 0.7 in the population, insertions were defined as homozygous (coverage frequency ≥0.7) or segregating at variable allele frequencies (coverage frequency <0.7). **B-C.** *P*-element insertions (rows) with homozygous (B) or variable allele frequencies (C) in *Harwich* that were detected (grey squares) or not detected (white squares) in the 40 PGC genomes (columns). Black crosses indicate coverage depth at the locus was insufficient to detect the insertion (<2 reads). Light grey shaded area represents the *'white'* X-chromosome of dysgenic males, which lacks *P*-elements. Magenta arrowhead indicates a locus containing two adjacent insertions on chromosome 2R in *Harwich,* which were rarely detected in PGCs. The *nos-moe::EGFP* transgene, which is flanked by short *P*-element sequences, was misidentified by the TE detection tool as variably segregating in *Harwich* (green arrowhead). **D.** Discovery rate of *Harwich P*-element insertions in PGCs,

expressed as the coverage frequency of insertions in *Harwich* against the proportion of PGCs in which the insertion was detected. Black line represents linear regression. r, Pearson correlation coefficient. **E.** Coverage frequencies of *Harwich* insertions detected in PGCs (left panel). Insertions on the X-chromosome detected in (hemizygous) male non-dysgenic PGCs are shown separately (right panel). Dashed line represents median, dotted lines represent first and third quartiles.

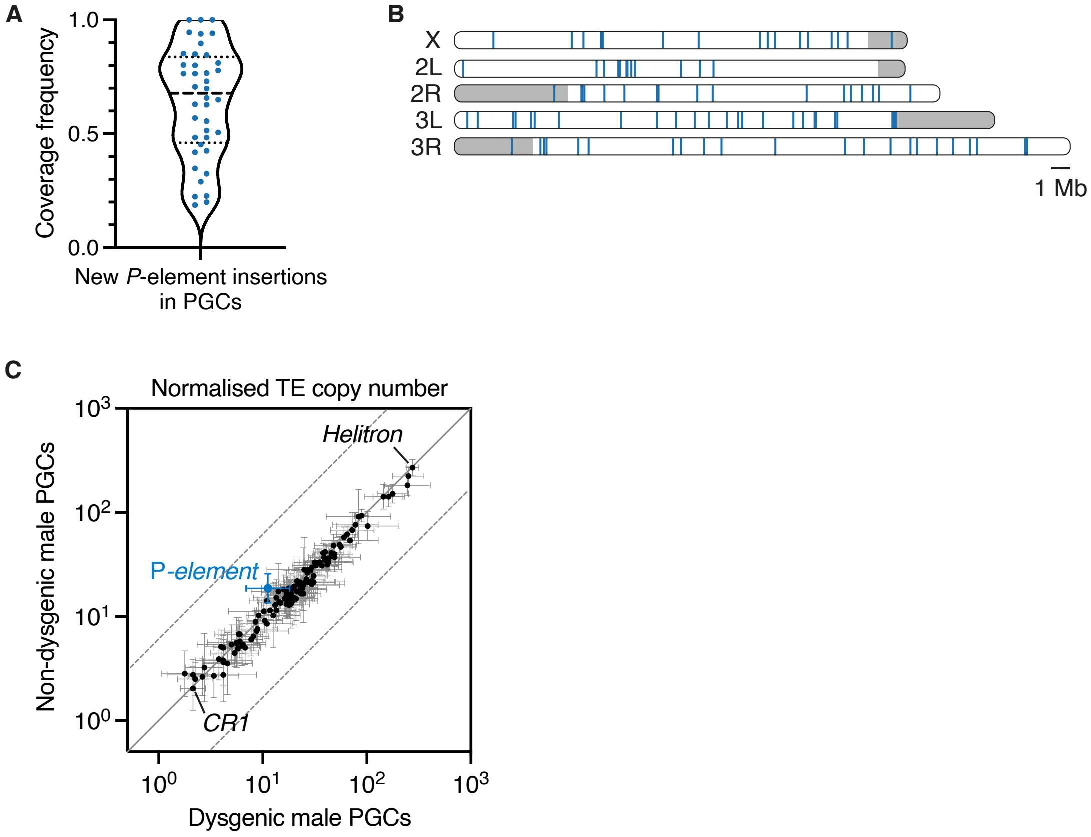

Supplemental Figure S5. Zygosity and genomic distribution of new *P*-element insertions in PGCs. A. Coverage frequencies of new *P*-element insertions (blue data points) detected in PGCs. Dashed line represents median, dotted lines represent first and third quartiles. **B.** Chromosomal distribution of new *P*-element insertions (blue lines) identified in PGCs. Grey shaded regions represent pericentromeric heterochromatic regions. **C.** Scatterplot of normalised genomic copy number for the *P*-element (blue) and 125 other TE families (black) in dysgenic versus non-dysgenic male PGCs. Solid grey line represents perfect correlation. Dashed grey lines indicate 5-fold difference. Error bars are ± one standard deviation.

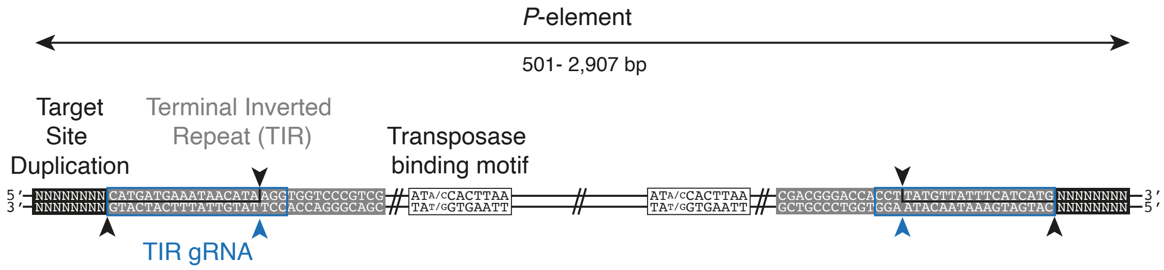

Supplemental Figure S6. An engineered Cas9 system to induce DSBs at *P*-element TIRs. Schematic showing *P*-transposase cleavage sites (black arrowheads) within 31-bp *P*-element TIRs (grey boxes) at the *P*-element 5' and 3' ends. The TIR-gRNA sequence (blue box) is followed by a PAM (TGG). The Cas9 cleavage site is 3 bp upstream of the PAM (blue arrowheads).

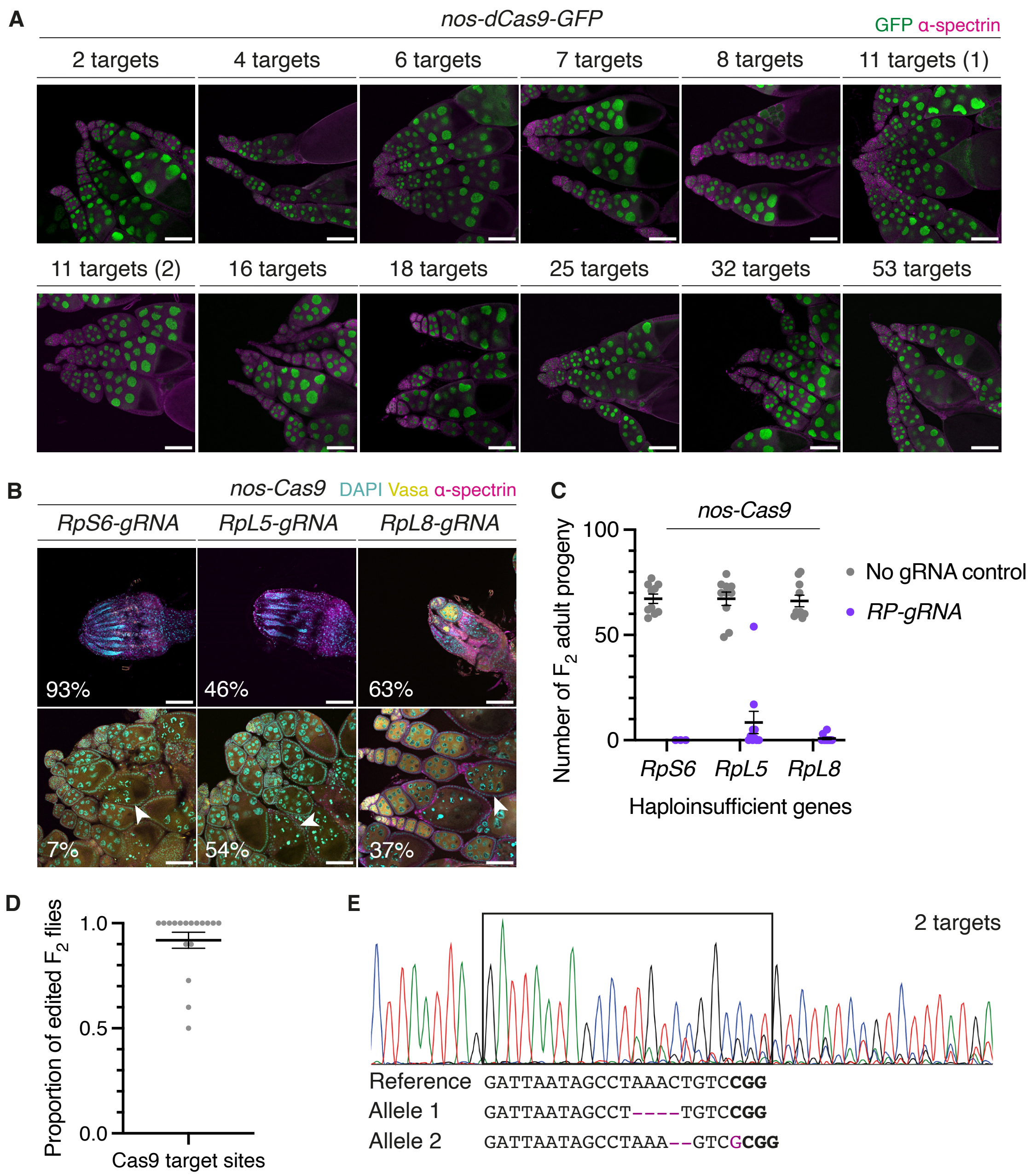

Supplemental Figure S7. High efficiency of DSB formation at Cas9 target sites in the germline. **A**. Ovaries of F_1 progeny from crosses between gRNA-expressing females and males expressing a GFP-tagged Cas9 variant that binds but does not cleave DNA in the germline (*nos-dCas9*), labelled with GFP (green) and α-spectrin (magenta). **B.** Ovaries of F_1 progeny from individual crosses between RP-gRNA-expressing females and *nos-Cas9* males, labelled with DAPI (cyan), Vasa (yellow) and α-spectrin (magenta). Percentages represent the relative proportion of progeny with rudimentary ovaries (upper panels) or ovaries with germaria and egg chambers (lower panel). White arrowheads indicate degenerated mid-stage egg chambers with aberrant nuclear morphology. **C.** Fertility tests (n = 10) of adult F_1 progeny in (B) with wild-type males. Black line is mean, error bars are ± SEM. $p < 0.0001$ for each pairwise comparison, unpaired *t*-test. **D.** Proportion of F_2 individuals (n ≥ 10) expressing 2-, 4-, 6-

, 7-, 8- or 11(1)-target gRNA (shown in Fig. 4C) harbouring DSB repair products at a given Cas9 target site (grey data points). Black line is mean, error bars are ± SEM. **E.** Sanger sequencing trace showing DSB repair products on the two alleles at the site targeted by 2-target gRNA (relative to the reference sequence). Inserted and deleted bases are shown in magenta. Scale bars, 100 µm.

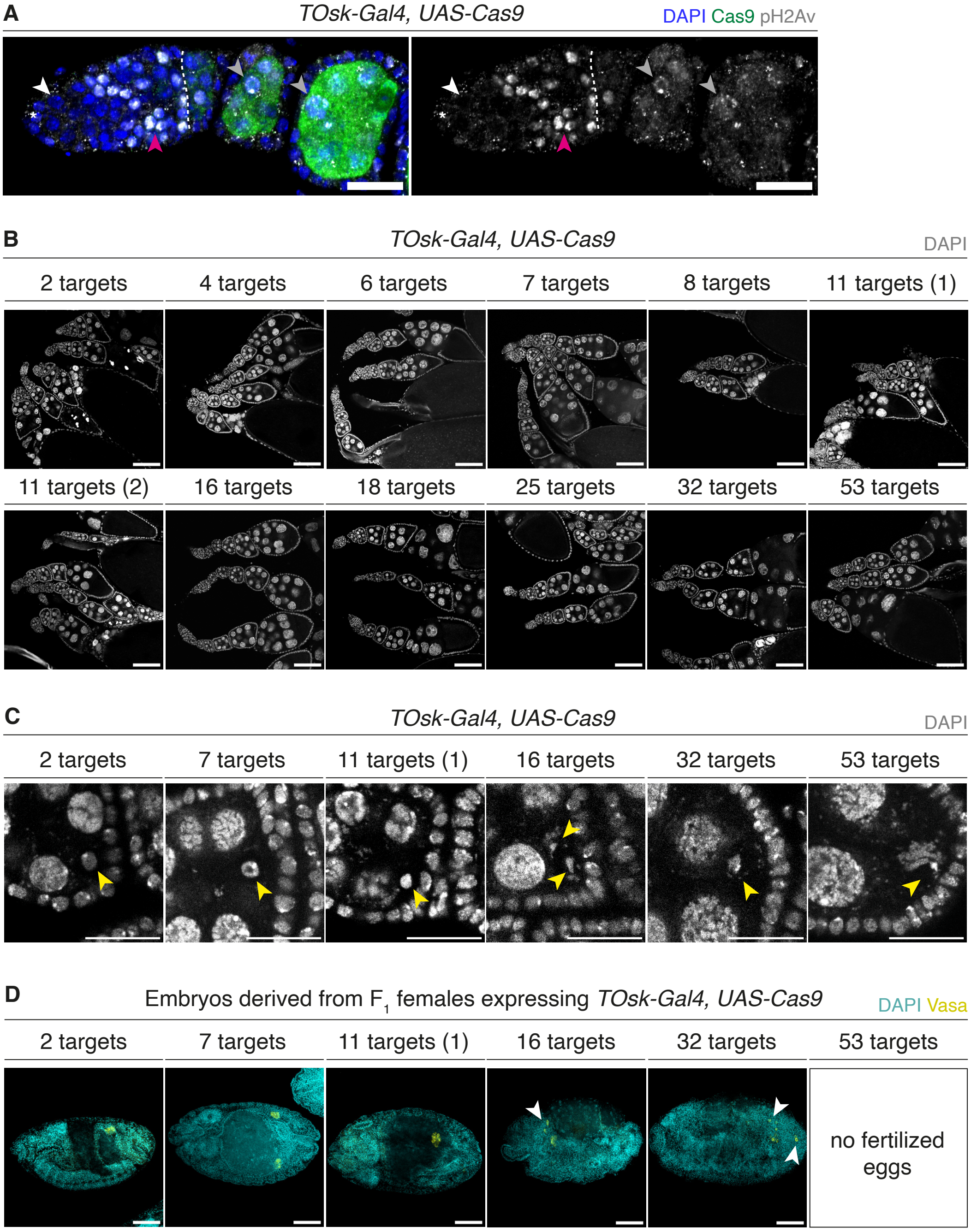

Supplemental Figure S8. Inducing DSBs in post-mitotic adult germ cells affects karyosome morphology and embryonic development of the F$_1$. A. Germarium from strain expressing Cas9 in the *TOsk* domain (demarcated by dashed line), labelled with DAPI (blue), Cas9 (green) and pH2Av (grayscale). Images are three-channel overlay (left) and single-channel (pH2Av, right). Asterisks indicate GSC niche, white arrowheads indicate GSCs, magenta arrowheads indicate meiotic germ cells and grey arrowheads indicate pH2Av-positive nuclei in early egg chambers. **B.** Adult ovaries of F$_1$ progeny from individual crosses between gRNA-expressing females and *TOsk-Gal4, UAS-Cas9* males, labelled with DAPI (grayscale). **C.** Karyosome morphology of oocytes (yellow arrowheads) in mid-stage egg chambers from ovaries in (B), labelled with DAPI (grayscale). **D.** Eggs laid by progeny in (B) aged

to ~16 hours, labelled with DAPI (cyan) and Vasa (yellow). White arrowheads indicate mislocalised PGCs. Scale bars, 20 µm (A and C) or 100 µm (B and D).

Supplemental Table Legends

Supplemental Table S1. *P*-element insertions in the *Harwich* strain.

Supplemental Table S2. New *P*-element insertions detected in dysgenic and non-dysgenic hybrid PGCs.

Supplemental Table S3. *P*-element copy number in wild-derived and transgenic strains.

Supplemental Table S4. Genomic copy numbers of gRNA sequences in the *nos-Cas9*, *nos-int;attP2* and *w;TM2/TM6* strains.
